# Innovative Treatment Strategies to Accelerate Wound Healing: Trajectory and Recent Advancements

**DOI:** 10.3390/cells11152439

**Published:** 2022-08-06

**Authors:** Praveen Kolimi, Sagar Narala, Dinesh Nyavanandi, Ahmed Adel Ali Youssef, Narendar Dudhipala

**Affiliations:** 1 Department of Pharmaceutics and Drug Delivery, School of Pharmacy, University of Mississippi, Oxford, MS 38677, USA; 2 Department of Pharmaceutical Technology, Faculty of Pharmacy, Kafrelsheikh University, Kafrelsheikh 33516, Egypt

**Keywords:** wound healing, chronic wounds, improved wound management, stem cells, nanotherapeutics, 3D bioprinting

## Abstract

Wound healing is highly specialized dynamic multiple phase process for the repair of damaged/injured tissues through an intricate mechanism. Any failure in the normal wound healing process results in abnormal scar formation, and chronic state which is more susceptible to infections. Chronic wounds affect patients’ quality of life along with increased morbidity and mortality and are huge financial burden to healthcare systems worldwide, and thus requires specialized biomedical intensive treatment for its management. The clinical assessment and management of chronic wounds remains challenging despite the development of various therapeutic regimens owing to its painstakingly long-term treatment requirement and complex wound healing mechanism. Various conventional approaches such as cell therapy, gene therapy, growth factor delivery, wound dressings, and skin grafts etc., are being utilized for promoting wound healing in different types of wounds. However, all these abovementioned therapies are not satisfactory for all wound types, therefore, there is an urgent demand for the development of competitive therapies. Therefore, there is a pertinent requirement to develop newer and innovative treatment modalities for multipart therapeutic regimens for chronic wounds. Recent developments in advanced wound care technology includes nanotherapeutics, stem cells therapy, bioengineered skin grafts, and 3D bioprinting-based strategies for improving therapeutic outcomes with a focus on skin regeneration with minimal side effects. The main objective of this review is to provide an updated overview of progress in therapeutic options in chronic wounds healing and management over the years using next generation innovative approaches. Herein, we have discussed the skin function and anatomy, wounds and wound healing processes, followed by conventional treatment modalities for wound healing and skin regeneration. Furthermore, various emerging and innovative strategies for promoting quality wound healing such as nanotherapeutics, stem cells therapy, 3D bioprinted skin, extracellular matrix-based approaches, platelet-rich plasma-based approaches, and cold plasma treatment therapy have been discussed with their benefits and shortcomings. Finally, challenges of these innovative strategies are reviewed with a note on future prospects.

## 1. Introduction

Skin accounts for 16% of the total body weight covering an average surface area of 1.85 m^2^ and represents the largest organ of the human body. In addition to playing a pivotal role in maintaining homeostasis, skin also acts a protective barrier against the external environment to prevent infections and fluid losses [1,2]. Therefore, the preservation of skin integrity is of utmost requirement to stay healthy. Skin injuries or wounds that are produced by trauma, burns, chronic diseases, and surgery may lead to disability and distress and pose a huge challenge on healthcare systems globally. Skin wounds are classified as acute (healing) and chronic wounds (non-healing) based on the consequences and underlying causes. Small acute wounds are repairable and generally healed through the skin’s own self-healing ability. The healing of skin is mediated by different dynamically coordinated cellular responses, extracellular matrix remodeling, and growth factors [3,4]. The healing process restores the functional and anatomical integrity of skin. However, large skin injuries and chronic wounds are slow healing, which are difficult to heal due to their susceptibility towards infections and fluid losses. Nevertheless, chronic wounds and large skin wounds impairs the skin’s functional and structural integrity, affecting millions of people (~8.2 million people) and thus posing immense economic and social challenges to healthcare systems globally [5,6]. For example: the mortality rate of chronic diabetic foot ulcers (DFUs) accounts for 30.5%, which is comparable to the mortality rate of cancer (31%) as per 5-year mortality rate statistics [7]. As per earlier reports, the management of chronic wounds accounts for over USD12 billion annually which are expected to rise to USD35 billion by 2023 [8,9,10]. The prevalence of chronic wounds or “silent epidemic” is further aggravated by diabetes, obesity, and an aging population. Moreover, different types of chronic wounds such as pressure ulcers, diabetic ulcers, and vascular ulcers take a very long time to heal due to aging, diabetes, or inappropriate treatment and are generally associated with the increased morbidity and mortality [11]. Despite the intense investigation, numerous strategies and advancements in skin wound healing, management of large wounds, and chronic wounds still remains an unmet therapeutic area due to difficulty in its assessment and wound care management [11,12]. Therefore, the development of improved and innovative strategies for skin wound healing holds profound global medical importance in the healthcare domain.

Currently, effective and quality wound healing pose huge challenges to clinicians as well as to researchers and incurs considerable costs. This can be substantiated by the growing global wound care products market which was about USD12 billion in 2020 and expected to reach USD18.7 billion by 2027 [13]. In general, skin wound therapies are classified into either “Conventional” or “Regenerative”. In conventional wound therapies, healing includes infection control using dressing changes (twice daily) and debridement of damaged tissues on weekly basis. The conventional treatment methods for large or full thickness skin defects includes split thickness skin autografts. Although the autografts are lifesaving, the requirement of multiple surgical interventions, limited donor sites availability, hypertrophic scarring, and possible functional alterations are associated shortcomings. On the other hand, regenerative wound healing employs emerging biomedical research technologies viz., smart wound dressings, bioactive biomaterial matrices, stem cells therapy, gene therapy, targeted drug/growth factor delivery, and bioengineered skin grafts to restore the pristine function of skin after healing and the reestablishment of damaged skin tissues [1,14]. This regenerative therapeutic approach allows improved and quality healing of wounds without scarring. An earlier wound repair approach has focused on layer-by-layer skin structure regeneration to two major compartments, epidermis, and dermis using skin substitutes involving bioengineered scaffolds or hydrogels that are encapsulated with cells. Cell-laden matrices that are encapsulated with keratinocytes, fibroblasts, or different types of stem cells have indicated promising results in accelerating wound healing with reduced scar formation [15,16]. Although this approach is promising as it mimics the biological and morphological features of skin, ample scope remains to improve the skin substitutes in terms of cellular composition and spatial distribution to recapitulate complex microarchitecture of native skin tissues [17]. Therefore, the current focus is mainly towards the development of various emerging and innovative strategies to accelerate wound healing without compromising the functional properties. Among all these emerging treatment modalities, stem cell-based therapies using different types of stem cells such as mesenchymal stem cells and induced pluripotent stem cells (IPSCs) have gained much interest as a part of stem cell-based regenerative medicine and have been tested in preclinical and clinical trials [18]. However, the poor stem cell viability in the wound region after implantation/grafting due to a harsh inflammatory environment limits the therapeutic potential of stem cell-based therapy [19]. In order to overcome this problem, improvement in the survival of stem cells and their biological functions, cell engineering via genome editing and reprograming of stem cells through genetic modifications presents exciting strategies for patient-specific wound therapy in patients with chronic wounds [20,21].

The therapeutic strategies that are being employed for wound healing in recent years based on wound types and intrinsic regenerative capacity include modern wound dressings (hydrogels dressings) [22], topical drug and growth factor delivery [23], hyperbaric oxygen therapy [24], auto/allograft and xenograft [14,25], cell-based therapy and engineered skin graft [26,27], vacuum-assisted closure [28], electrotherapy [29], negative-pressure therapy [30], ultrasound [31], and exosome-based approach [32]. Although these treatment modalities are easy to use and well-studied, there still remains various limitations and disadvantages. Therefore, cost effective and non-invasive wound care therapy development is pertinent with regard to quality wound healing. Moreover, the ultimate goal of wound management is to provide better tools and technology for diagnosis and prognosis for a personalized treatment plan. In the quest for improved wound therapies, various new treatment strategies and approaches have been explored for wound healing in last decade such as nanotherapeutics, stem cell therapy, 3D bioprinting, and skin organoids and on-a-chip technologies [33,34,35,36]. This review article provides a comprehensive overview of the recent advancements in wound healing therapies using various emerging and innovative approaches. Herein, we briefly discuss the skin anatomy and wound healing process followed by conventional methods that are being utilized for wound healing with advantages and shortcomings. Furthermore, we have extensively reviewed the new emerging and innovative strategies based on new technologies for wound healing with benefits and recent advancements. The different emerging strategies that were reviewed include nanotherapeutics approaches using different nanomaterials, 3D bioprinting, stem cell therapy, ECM-based therapy, cold plasma therapy, and platelet-rich plasma (PRP) therapy. Finally, we present the challenges and future prospects of modern wound healing therapies.

## 2. Skin Architecture and Functions

**Figure 1:**
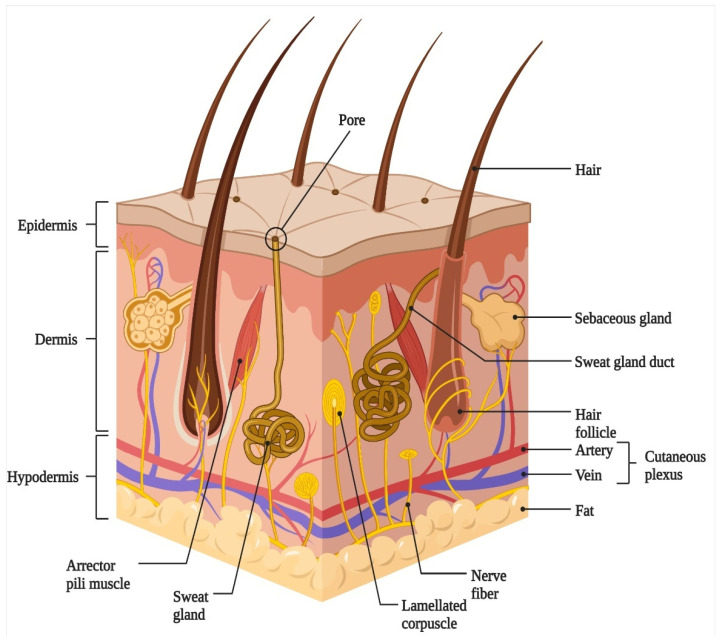
Schematic representation of basic human skin anatomy depicting the different skin layers and their components.

The skin represents the largest organ of human body and is comprised of three distinct layers: the epidermis, dermis, and hypodermis along with more than 50 different cell types interspersed within anisotropic collagenous ECM [37]. The main functions of skin include proper protection against extremal factors (ultraviolet light, microbes, chemical, thermal etc.), the prevention of water loss from the body, regulation of body temperature, and facilitating metabolic functions (synthesis of vitamin D). The epidermis and dermis present two main structural layers of skin which are connected with the basement membrane. The third layer, the hypodermis, is a subcutaneous layer that lies below these layers along with adipose tissue (Figure 1). The epidermis, an avascular keratinized stratified squamous epithelium forms the outermost waterproof layer of skin that plays a pivotal role in controlling the moisture into the body [38]. The epidermis layer is generally nourished through the diffusion of intercellular fluids that are derived from the dermal vasculature [39]. The epidermis layer comprises of several specialized cells such as keratinocytes, Merkel cells, melanocytes, and antigen-presenting dendritic Langerhans cells [40]. Keratinocytes are predominant epidermal cells that are present in the epidermis layer which accounts for approximately 90% of the population of cells. Among the cell types that are present in epidermis layer, keratinocytes being the primary cells are mainly involved in epidermal healing function, and water transport. A variety of currently identified growth factors, chemokines and cytokines are secreted by keratinocytes for the regulation of inflammatory and immune responses in pathological skin conditions [41]. The non-epithelial cells, Merkel cells, relay the touch information in terms of texture and pressure to brains. Melanocytes secrete skin pigment, melanin, which are received by keratinocytes. Langerhans cells secrete antimicrobial peptides that support the innate and adaptive immunity [26]. 

The epidermis further consists of the stratum corneum, stratum granulosum, stratum spinosum, stratum lucidum, and stratum basale. Basically, keratinocytes are generated from stem cells that are present in the basal layer of skin which differentiates up to 3–6 weeks and gets transformed into corneocytes and ultimately forms stratum corneum layer [42]. Keratinocytes synthesize many proteins including keratin which plays a major structural role in the stratum corneum. In addition to proteins, cell membranes and complex lipids that are present in stratum corneum provide a major protective barrier against microbes and dehydration. After approximately every 28 days, the migration of fully differentiated cuboidal basal keratinocytes containing large nuclei, phospholipid membrane, and organelles begins from the basal layer [43]. During this turnover process, an enhanced accumulation of keratin and lipids occurs which undergoes terminal differentiation to become stratum corneum. The epidermal layer and dermal layer are separated from each other by a basement membrane termed as dermo-epidermal junction, wherein the cell adhesion molecules hemidesmosomes are attached. The complex dermo-epidermal junction structure is comprised of keratinocytes from the basal layer, ECM components, basal lamina, filaments, and anchoring fibrils [44]. During the wound healing process, restoration of the dermo-epidermal junction is also required in order to avoid the risk of epidermis separation from the dermis layer [45].

The dermis, the inner layer of skin, is present below the epidermal layer and above the hypodermis and forms approximately 90% of the skin weight [46]. The dermal layer is a connective tissue and is comprised of abundant ECM, vascular endothelial cells, fibroblasts, Schwann cells, mast cells, sweat glands, blood vessels, hair follicles, and free and encapsulated nerve endings [47,48]. The dermal layer consists of two layers: the superficial (papillary dermis) layer and the deep (reticular dermis) layer that is populated with fibroblasts and thick collagen fibers, respectively [49]. Fibroblasts are the main dermal cells which secrete glycosaminoglycans, collagen, and elastin and are responsible for providing elasticity and mechanical strength to the skin [49]. The dermal layer which accommodates the neural, vascular, and lymphatic of skin provides mechanical and metabolic support to the epidermal layer owing to its structural features. Under the dermis, the subcutaneous tissue layer is the hypodermis which connects the dermal layer of skin with other structures viz., muscles or bone [50]. The hypodermis layer is comprised of loose connective tissue, adipocytes, proteoglycan- and glycosaminoglycan-absorbing fluid, blood vessels, and nerves [51]. The major functions of the hypodermal layer include fat storage and the regulation of body temperature. 

## 3. Wounds and Wound Healing Process

Wounds are defined as injuries or any disorders that compromises the skin integrity to skin structure that are caused by extrinsic factors (cuts, burns, pressure), surgery, or pathological conditions such as diabetes or vascular diseases [4]. Wounds are classified into two types: acute or chronic wounds on the basis of healing duration and healing process. Acute wounds are assorted injuries that are caused by various mechanisms such as radiation, extreme temperature changes, chemicals etc. Acute wounds are further classified into several types according to their size and depth as superficial, deep dermal, or full thickness wounds [52]. The acute wounds generally heal in 4–12 weeks’ time with a restoration of functional and anatomical skin integrity through an organized traditional wound healing process [53]. In contrast, chronic wounds are difficult to heal wounds due to previous pathological conditions such as diabetes, autoimmune diseases, or venousstasis. In addition, prior infection, inflammation, tumors, or different physical agents may also lead to chronic wounds. The characteristics of chronic wounds include low mitogenic activity, higher levels of cytokines and proteases, low secretion of growth factors, over-production of reactive oxygen species (ROS) and matrix metalloproteases (MMPs), suppressed angiogenesis, fibrosis, and ECM destruction [54,55].Unlike acute wounds, the healing process is delayed (more than 12 weeks) in the case of chronic wounds, that further paves the way for infections [56]. The delayed healing process in chronic wounds further prevents optimal and functional restoration of skin integrity. Therefore, the development of newer approaches and strategies are very much needed to accelerate the wound healing process, particularly in the case of chronic wounds.

**Figure 2:**
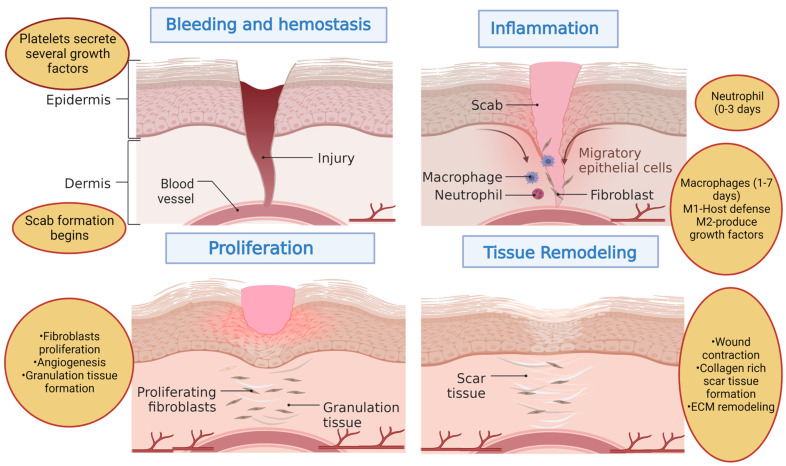
Different stages of wound healing. All the four phases with highlighted key events play a pivotal role in cutaneous wound healing.

Wound healing is a complex and dynamic intricate physiological process which is divided into four interrelated and overlapping phases: homeostasis, inflammation, proliferation, and tissue remodeling phases as shown in Figure 2 [57]. After acute injury, a highly coordinated cascade of events initiates the wound healing process that co-ordinates the progression of wound healing through the abovementioned overlapping but distinct phases of healing [58]. A series of tightly regulated events involving the cellular release of various growth factors (GFs) and cytokines, chemotaxis, phagocytosis, collagen remodeling with neo collagen synthesis, and collagen degradation occurs within the distinct wound healing phases [59]. The healing process in adults is comparatively slower than infants and often results in scarring (fibrosis healing) rather than regeneration, this further necessitates the need for innovative strategies for scarless quality wound healing. The healing process generally stops and fails to accurately repair skin tissue in the case of chronic or a non-healing wounds due to the large wound size or depth of wounds which leads to keloid formation with undesirable scars [60]. The initial phase of wound healing immediately after injury is hemostasis wherein the body attempts to stop bleeding through vascular constriction and scab formation involving platelet aggregation [61]. The platelets arrive at the wound site and promote blood coagulation without disrupting normal blood flow. This blood clotting provides temporary wound sealing and thus avoids unnecessary blood loss. This process also limits the spread of microorganisms post-injury [37,62]. The damaged epithelial and endothelial cells and blood clotting releases a variety of chemotactic factors which attracts the inflammatory cells to initiate the next phase of healing. Hemostasis is followed by the inflammation phase wherein immune cells such as neutrophils, microphases, monocytes, and lymphocytes migrate to the wound site in response to damage signaling factors to prevent microbial infection. In this phase, the release of several growth factors and cytokines such as platelet-derived growth factor (PDGF), fibroblast growth factor (FGF), epidermal growth factor (EGF), and transforming growth factor (TGF); interleukin (IL)-1, IL-6, and IL-17; tumor necrosis factor (TNF); along with ROS are evident in addition to the recruitment of different immune cells [63]. During the inflammation phase, the migration of different white blood cells occurs through the bloodstream to reach the wound site for further engulfment and digestion of microbial contaminants [64]. The increased permeability and dilation of the capillaries mediates the migration of the white blood cells towards the wound site. Neutrophils, which play an antimicrobial role, arrive at the wound site first and lie there for 24 h after triggering the wound healing process. This is followed by the recruitment of other immune cells such as macrophages and lymphocytes that are mediated by secreted growth factors (vascular endothelial growth factor) and cytokines of neutrophils (interleukins-17) for the continuous digestion of bacteria and remaining debris for skin reconstruction [63,65]. The duration of the inflammation phase in wound healing is generally 1–4 days post-injury that prepares the wound for regeneration through essential phagocytosis and debris removal process [61,66]. In the late stage of inflammation, a switch of macrophage phenotypes from a pro-inflammatory (M1 phagocytic) to anti-inflammatory (M2 pro-regenerative) along with neutrophil apoptosis occurs [67,68]. The growth factors and cytokines that are produced by the immune cells during the inflammation phase recruit various cells such as stem/progenitor cells, fibroblasts, keratinocytes, and endothelial cells to initiate the proliferation phase [69]. During this phase the skin tissue reconstruction starts through the fibroblasts (ECM production to form granulation tissue), proliferation and migration of endothelial cells (vascularization), and restoration of an epidermal barrier through wound re-epithelialization [37,70]. 

Fibroblasts proliferate and form granulation tissue by producing ECM components such as proteoglycans, hyaluronic acid, procollagen, and elastin that acts as a suitable substrate for new blood vessel growth. The growth of new blood vessels through endothelial cells provides oxygen and nutrient supply to the cells. The chemicals that are produced by granulation tissue tend to degrade the existing blood clot, and collagen synthesis by the fibroblasts provides mechanical strength to the wound. The differentiation and proliferation of keratinocytes leads to re-epithelialization to provide a superficial epidermal cover to the wound [71]. Moreover, cellular crosstalk between the different skin cells promotes extracellular matrix (ECM) deposition, angiogenesis/re-vascularization keratinization, and re-epithelialization [61]. This stage is followed by the remodeling or maturation phase wherein the granulation tissue is replaced with collagen type 1 synthesis and the generation of various skin appendages (sweat glands, sebaceous glands, hair follicles) to restore the functional restoration of the wounded area to its original pre-injury state [37] In this phase, fibroblasts differentiate into myofibroblasts, and collagen type III is changed to collagen type I for providing superior tensile strength to the skin [72]. The remodeling of the ECM is important as excess collagen deposition and modulation of matrix metalloproteinases (MMPs) activity leads to dysregulated wound healing with the formation of hypertrophic scars [73,74]. Wound healing is achieved after the completion of all these phases in the case of acute wounds. However, chronic wounds fail to complete all these phases and impairs the wound healing process. Hypoxia and altered cellular phenotypes are characteristics of chronic wounds and all the wound areas are not in same phases of the wound healing process [18,75]. The hallmark of chronic wounds includes persistent inflammation which generally fails to progress to other wound healing process phases due to differences in their etiology at the molecular level compared to acute wounds. This leads to the excessive recruitment of inflammatory cells and the release of various growth factors to the wound bed leading to a long inflammatory phase in chronic wound healing [59]. In addition, growth factors and vascular insufficiency further leads to delayed wound healing in chronic wounds. As a result, wound lesions in chronic wounds indicate elevated levels of proteases because of the activation of pro-inflammatory cytokine cascade for a prolonged period of time which is otherwise tightly regulated in acute wound healing. Taken together, a systematic approach is a prerequisite for the assessment and treatment of chronic wounds. 

## 4. Conventional Approaches Employed for Wound Healing

The cost of wound treatment currently accounts for USD12 billion annually, and is expected to reach USD35 billion in 2023 [10]. Therefore, the treatment modalities for wounds should be efficient and cost effective in order to reduce the mortality and financial burden. Acute wounds generally follow the normal wound healing process and results in expected and organized skin tissue repair after the completion of all the phases of wound healing [76]. However, the treatment of hard to heal or chronic wounds follows the ‘TIME’ guidelines, wherein this acronym stands for stands for tissue (T) assessment and debridement of nonviable tissues, infection/inflammation (I) control and etiology assessment, moisture balance (M), and epithelial edge (E) advancement as it takes a painstakingly long time to heal and incur immense cost to treatment [77]. Nowadays, the new approach of triangle of wound assessment (TWA) considers the peri-wound skin as an integral part of the treatment regimen along with the wound bed and wound edge [78]. Moreover, a systematic approach is required for the assessment and treatment of chronic wounds regardless of their etiologies. The conventional wound management practices include various surgical procedures, non-surgical therapies, and pharmacological agent-based treatment regimens for skin wounds repair [79]. The surgical treatment procedure for wound healing includes debridement, skin grafts, and skin flaps, while non-surgical therapies include topical formulations, wound dressings, and skin substitutes. After the initial wound volume assessment and cleaning of the wounds, the debridement process starts [80]. Surgical debridement of devitalized tissue prepares the wound bed in order to accelerate ECM remodeling and avoid wound healing impairment [81]. Therefore, surgical debridement remains the gold standard in wound management owing to its effectiveness [82]. This surgical procedure is fast and effective and is performed under anesthesia.; however, the increased risks of the general anesthesia, and damage to surrounding tissues are associated risks with this process. 

Other surgical treatment procedures for wound management includes skin grafts that are based on the type of wounds and etiology. Skin grafts are utilized in the case of wounds with large tissue loss and chronic wounds and represents the most common surgical alternative treatment for wounds. Based on the thickness of the skin grafts, they are classified into split-thickness skin grafts and full-thickness skin grafts. Split-thickness skin grafts are regarded as surgical standards for covering small wounds with epidermal damage only. In this grafting, mainly the epidermis and a part of the dermis is harvested from the healthy skin and utilized to cover the damaged skin area. However, this grafting method is not useful for patients wherein the wounds coverage area is more than 30% of the total body. Other shortcomings include pain, itchiness, and increased contraction during the healing process that leads to scarring. In contrast, full thickness skin grafts are useful for large and deeper wounds as it includes both the healing of the epidermal and dermal layers. These grafts provide better healing as less contraction is there and it results in scarless repaired tissue. However, restricted supply of donor skin and the requirement of a vascularized wound bed limits the full thickness grafts usage. Furthermore, skin grafts are classified into three types viz., autografts, allografts, and xenografts that are based on the donor origins [83]. Autografts remain the surgical gold standard for skin repair as healthy skin is taken from the same patient and transplanted to the injured area for restoration of skin integrity [84]. The advantages of using autologous tissue in autografts include no immune rejection and the restoration of local vasculature and epidermal function. However, a painful healing process, insufficient normal skin sites, and consequent scarring are few of the limitations that are associated with the utilization of autografts. 

Allografts represent an alternative to autografts, wherein the donor skin is from a different person. The advantages of allografts include the immediate availability of donor skin and prolonged storage for future usage. On the other hand, xenografts are skin grafts which are obtained from different species and transplanted into humans. For example, porcine skin grafts have been utilized in burn wounds healing wherein the burns are under 30%. Allografts and xenografts are associated with limitations such as immune rejection, the risk of disease transmission, painful healing, and scarring. 

Conventional non-surgical therapies for wounds include different types of wound dressings, topical formulations, scaffolds/hydrogels-based skin grafts, and skin substitutes. These non-surgical therapies debride the wound bed, correctly balance the moisture, manage infection and inflammation, and control re-epithelialization and contraction. The topical formulations route is the most common route of drug administration to skin and includes gels, emulsions, pastes, creams, foams, lotions, and sprays etc. Topical antibiotics in the form of creams and ointments containing neomycin, povidone-iodine, silver sulfadiazine, and others are effective against a broad range of bacterial infection preventions. The topical antibiotics are more effective in the inflammation phase of wound healing wherein the risk of infections is higher. Antibiotic topical application should be discontinued afterwards otherwise it may lead to hypersensitivity and allergic reactions. Other ointments formulations containing collagenase and dexpanthenol have been utilized for improving the healing process as it controls the proliferation of fibroblasts, accelerates re-epithelialization, and ECM remodeling [85]. In recent years, growth factors that are based topical formulations have been tested in wound healing due to their beneficial effects. However, limited bioavailability of growth factors due to its rapid clearance from the wound site restricts its utilization. In order to alleviate this shortcoming, emerging approaches using nanoparticle-encapsulated growth factors with enhanced stability and bioavailability are topically applied to the wound area. In a recent study, the topical application of microspheres containing fibroblast growth factor (FGF-10) reported improved collagen synthesis and angiogenesis [86].

Other non-surgical therapies include wound dressings for improved wound healing. Wound dressings maintain a humid environment and provides a high permeability to oxygen, which is a prerequisite for quality wound healing. The most common wound dressings include dressings incorporating various antimicrobial agents for the treatment of acute wounds. Currently >3000 types of wound dressings are available on the market which are classified into biological, artificial, and traditional wound dressing products on the basis of the cause and type of wound [87]. The most common traditional and modern wound dressings being utilized in wound healing include cotton gauze dressings, human amniotic membrane, transparent polymeric film dressings, foam/sponge-based wound dressings, hydrocolloid dressings, polymeric wound dressings, alginate-based wound dressings, hyaluronic acid-based dressings, chitosan and chitin-based dressings, hydrogel-based wound dressings, nanofiber-based wound dressings, microbial cellulose-based dressings, antimicrobial dressings, cell-based dressings etc. [88]. Among all these wound dressings, film-based, hydrogels-based, and polymeric wound dressings are the most extensively utilized for wound healing applications. Polymeric film-based dressings have been utilized for the last three decades with gradual improvement in the design and application. Film- based dressings are transparent and semipermeable and can easily conform to the wound surface. The advantages of film-based dressings include high vapor transmission rate, impermeability to bacteria, and automatic scab removal ability. Polymeric films that are derived from diverse natural and synthetic polymers have been utilized as wound dressings for abrasions, superficial wounds, infected wounds, surgical wounds, and burns. However, adhesiveness, lack of ability to absorb a high volume of wound exudates, and the need for frequent changes necessitates further improvement in film-based wound dressings. Currently, various FDA-approved film-based dressings are commercially available such as Biooclusive™, Tegaderm™, Hydrofilm/Hydrofilm™, Opsite™ etc., varying in permeability, adhesiveness, and conformability [89]. In the last decade, moist wound dressings that are based on hydrogels have garnered much attention for wound healing applications owing to various inherent advantages viz., high wound exudate absorption ability, moisture retention ability, and the ability to promote migration and proliferation of keratinocytes and fibroblasts. These hydrogel-based wound dressings reduce pain and provide necessary moisture through high absorbent ability and cooling effect. Moreover, hydrogel-based dressings are suitable for the treatment of dry wounds, superficial wounds, necrotic wounds, burns, deep wounds etc. With the advancement in biomaterials and design strategies, hydrogel-based dressings are now available as injectable hydrogels, self-healing hydrogels, hydrogels with drug/growth factor/antimicrobial agents release properties for improved wound healing [90,91,92].

Wound dressings are generally derived from natural and synthetic biomaterials and also incorporate growth factors, drugs, and other bioactive molecules. Wound dressings that are derived from natural biomaterials such as silk fibroin, alginate, hyaluronic acid, and other mimics the ECM environment of skin and accelerates the process of wound healing. In addition, composite wound dressings that are derived from natural, synthetic, organic, or inorganic biomolecules have also been utilized for the treatment of a wide range of wounds. With the advancement of nanotechnology, wound dressings incorporating nanoparticles, antibiotics, growth factors, and bioactive agents have improved the bioactivity of wound dressings. Currently, an improved approach, stimuli-responsive (pH, temperature, oxygen) wound dressings and 3D printed/bioprinted wound dressings are being extensively utilized for wound healing purpose.

Other most common improved conventional approaches for wound healing include bioengineered skin substitutes. Tissue engineered skin substitutes are comprised of three components viz., biomaterials, cells, and growth factors that mimic the normal skin and provides enhanced skin regeneration by providing a protective semi-permeable barrier around the wound area. A variety of natural biomaterials, growth factors, and cells are being explored for the development of skin substitutes. Skin substitutes reduce the chronic wound-associated mortality and morbidity without the requirement of a secondary surgical procedure unlike earlier conventional surgical methods. The other advantages of skin substitutes include its effectiveness and safety. There are three types of skin substitutes: epidermal skin substitutes, dermal skin substitutes, and epidermal-dermal skin substitutes that are based on the proportion of damage to the skin. The most common epidermal and dermal skin substitutes that are available on market include Integra, Dermagraft, Alloderm, Transcyte, Matriderm etc. These commercial skin substitutes are extensively utilized for the treatment of partial and full thickness wounds, chronic ulcers, or burns [93]. Epidermal skin substitutes are comprised of keratinocytes, growth factors, and a biomaterial scaffold for support. In contrast, dermal skin substitutes are comprised of fibroblasts, growth factors, and biomaterial matrix support for wound healing. However, epidermal, and dermal skin substitutes alone are not sufficient for some skin injuries wherein both the layers are damaged. Consequently, there is a requirement for more sophisticated cellular skin substitutes that mimic both the epidermal and dermal layers and provides extracellular matrix components and growth factors and cytokines for wound healing. Apligraf and OrCel indicate two commercial dermo-epidermal cellular substitutes which are being used for the treatment of both partial and full thickness burns, diabetic foot ulcers, and chronic wounds [94]. However, there still remains a few limitations such as the risk of tissue rejection and the high cost that is associated with the epidermal-dermal cellular skin substitutes, which further necessitates newer research studies and technology advancements to alleviate these problems. Moreover, the most common conventional wounds management wound dressings and grafts combined with newer technology such as bioengineered skin substitutes have been extensively utilized for improving wound healing. Another strategy for improved wound healing includes a combination of traditional cotton gauze with antibacterial nanoparticles. In addition, topical formulations that are combined with nanoparticles and nanostructured carriers have improved the treatment modalities for wound healing. In recent years, various emerging methods such as bioengineered skin substitutes, vacuum-assisted wound closure, stem cell therapy, and growth factor/cytokine therapy represent alternatives to traditional methods [88]. However, most of the abovementioned methods still lack assessment and effectiveness in large scale studies. Therefore, the improvement of conventional wound management through the exploration of emerging and innovative approaches is pertinent for cutaneous wound healing to fulfill patient needs in terms of effectiveness, cost-effectiveness, and cosmetic appearance. With recent advancements in science and technology and precision medicine, various emerging and innovative methods such as 3D bioprinting, cold plasma treatment, platelet-rich plasma-based treatment, and ECM-based approaches offer tremendous potential for improved quality wound healing. 

## 5. Innovative Strategies for Wound Healing

Currently, not a single treatment approach is sufficient for complete wound healing owing to differences in the molecular and cellular mechanisms of different types of wounds. Recent developments and advancements in technologies have substantially expanded the knowledge about the assessment and treatment strategies in the wound healing research domain. The paradigm shift from dry dressings to moist wound dressings, stimuli responsive wound dressings, growth factor-based therapy, tissue engineered skin, bioengineered human skin substitutes, gene therapy, nanotherapeutics, and stem cell therapy has revolutionized the development in this field. In addition, advancements in emerging technologies such as the 3D bioprinting field, platelet-rich plasma (PRP) therapy, and ECM-based approaches have paved way towards personalized wound healing. Nevertheless, the assessment and treatment of chronic wounds still poses a cause of concern. Therefore, newer and innovative treatment strategies are required for wound healing that consider effectiveness, benefit-to-risk, and the cost effectiveness. In this section, we will list and discuss a few emerging and innovative treatment strategies which are being employed and explored for wound healing along with advantages and challenges.

### 5.1. Nanotherapeutics-Based Strategies

**Figure 3:**
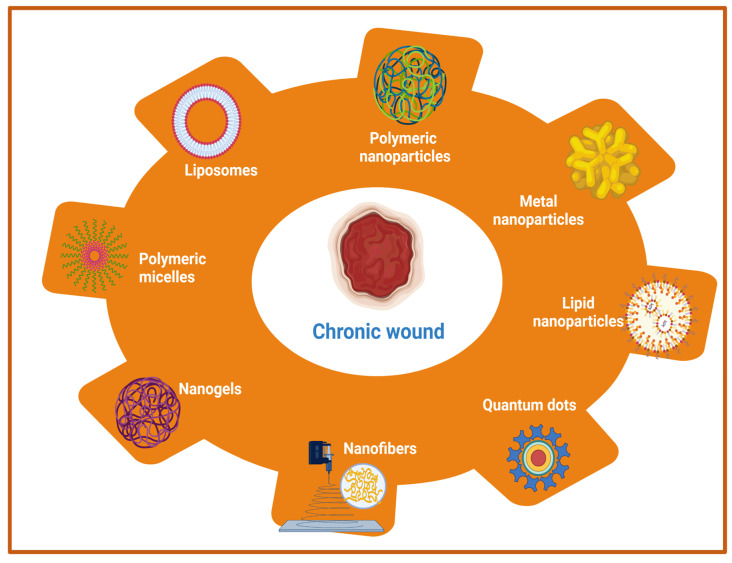
Schematic representation of nanotherapeutic approaches using a wide range of nanomaterials for chronic wound healing.

Owing to its complex pathophysiology and associated local and generalized complications, chronic wound management is far more challenging than acute wounds. Earlier approaches utilized the systemic administration of antimicrobial agents, antibiotics, and other local application of drugs in order to improve the wound healing of hard-to-heal wounds. However, these approaches are associated with various shortcomings such as low, or sub-optimal penetration of drugs into deeper skin tissues and the development of bacterial resistance with prolonged usage of antibiotics [95,96]. Therefore, the adoption of newer therapeutic modalities that can eradicate the risks of systemic microbial infection as well as augment drug delivery to the deeper tissues in chronic wounds is a prerequisite for quality wound healing. The emerging therapeutic options include nanotherapeutics, stem cell therapy, phototherapy, and different biological therapies such as microbiome therapy, reactive oxygen species (ROS), and NO generators. Among these emerging therapeutic approaches, nanotherapeutic-based strategies have demonstrated exceptional treatment efficacy for enhanced wound healing using different types of nanomaterials [97,98]. In the nanotherapeutics approach, different forms of nanomaterials (nanoparticles, nanofibers, nanogels, nanoemulsion) that are loaded with antimicrobial agents, antimicrobial peptides, growth factors, interferons, and others through the delivery at wound site have been employed for the treatment of different types of wounds. In addition, nanotechnology-based approaches demonstrated the potential to overcome the various obstacles that are associated with conventional wound healing modalities such as sepsis, sub-optimal penetration to deeper skin tissues, and delayed wound healing. The rapid development of nanotechnology in last two decades has opened new avenues for the delivery of drugs, antimicrobial agents, antibiotics, different biomacromolecules (proteins, peptides), growth factors, DNA/RNA, and different therapeutic moieties for chronic wound healing by playing a vital role in controlling microbial infection, inflammation, hemostasis, and promoting cellular proliferation [99,100]. Moreover, nanoparticulate drug delivery systems using different nanocarriers provide sustained and controlled delivery of a wide range of drugs/therapeutic agents at an optimal concentration with an extended half-life, improved bioavailability, improved pharmacokinetic profiles, and decreased drug administration frequency [101,102]. The therapeutic role of nanocarriers in wounds is attributed to its small size (ranging from 10 to 100 nm) and physicochemical properties, which allows intracellular drug delivery, maintenance of a moist environment with enhanced penetration, and degradation stability [33,103]. Additionally, high encapsulation efficiency of nanocarriers of different drugs and biomolecules increases the effectiveness of delivery for wound healing and skin regeneration applications [104]. Nanotherapeutic-based strategies provide several advantages for controlling the microbial infections in chronic wounds. The advantages include an improved half-life and bioavailability of drugs, improved penetration of drugs into bacterial biofilms and tissue barriers, enhancement of interactions between drugs and microbes, and the ability to increase the concentration of drugs at infection sites [105]. There are two main categories of nanomaterials that are being utilized with distinct properties and efficacy for the management of chronic wounds [98,102]. The first category includes organic nanomaterials which comprises of polymeric nanoparticles, nanogels, nanofibers, nano emulsions, micelles, liposomes, nanosheets, ethosomes, and solid-lipid nanoparticles. The second major category of nanomaterials include inorganic nanomaterials viz., metal nanoparticles, quantum dots, carbon nanotubes, and magnetic nanoparticles [106]. A list of different nanomaterials that are being utilized for wound healing applications are listed in Figure 3. 

Furthermore, nanoparticles in wound healing applications are classified into two categories (1) nanoparticles with intrinsic properties for acceleration of wound healing, and (2) nanoparticles as drug delivery systems. Moreover, nanoparticles can be bioengineered in such a way that they can be used either as a therapeutic device or as drug delivery vehicles for the sustained and controlled delivery of various therapeutic molecules for improved wound-healing processes. Owing to the presence of various advantages, metal nanoparticles, metal oxide nanoparticles, polymeric nanomaterials (nanoparticles, nanofibers, nano emulsion), and other nanotherapeutics have been widely employed for the treatment of chronic wounds. Metallic- and metal oxide-based nanoparticles such as silver, gold, zinc, zinc oxide (ZnO), aluminum oxide, titanium dioxide, iron oxide, copper oxide, and gallium possess outstanding antibacterial properties that help in the stimulation of wound healing [107,108,109]. The anti-bacterial activities are attributed to the production of ROS, inhibitory enzymes, damaging effects on bacterial DNA, and biofilms breaking ability to inhibit the bacterial growth [110]. In addition, other materials such as cerium, nitric oxide (NO) nanoparticles, bioactive glass, and carbon-based nanoparticles with intrinsic bioactivity have been utilized for chronic wound management [111]. However, toxicity that was induced by metal-based nanotherapeutics should be considered beforehand otherwise it’s in vivo applications would be restricted. In order to overcome this problem, the optimization of size and properties of metal nanotherapeutics would provide benefits in terms of reducing toxicity. Other than metal-based nanotherapeutics, polymeric nanomaterials with inherent biocompatibility and biodegradability are pushing the forefront for wound healing applications. Polymeric nanotherapeutics include polysaccharide-based polymers, protein-based polymers, polyester-based polymers, polyamide-based polymers, cationic polymers (chitosan), etc. The different types of polymeric nanomaterials include nanoparticles, nanofibers, nanoemulsion, nanosheets, nanogels, polymeric micelles, liposomes, and others for wound healing and skin regeneration [112]. Another crucial strategy using polymeric nanotherapeutics includes the encapsulation of antibiotics/growth factors within polymeric nanomaterials to prevent wound infection and thus shorten the wound healing time [113]. A comprehensive list of nanotherapeutic-based strategies using different organic and inorganic nanomaterials for the treatment of acute and chronic wounds are summarized in Table 1.

Another feasible nanotherapeutics strategy for the treatment of chronic wounds involves the regulation of the inflammatory state using different nanomaterials. In the case of burn wounds, mainly TNF-α, IL-1β, and IL-6 are overexpressed as inflammatory factors, while IL-18 is highly expressed in the case of diabetic wounds. Therefore, the careful regulation of the inflammation phase would be required as inflammatory markers vary in different types of chronic wounds. Silver nanoparticles and polymeric nanofibers demonstrated a reduction in the levels of inflammatory markers IL-1β and IL-6 but not IL-18, suggesting its poor anti-inflammatory effects against diabetic wounds [114,115]. Another limitation includes the difficulty in the identification of the inflammatory phase as the inflammation phase overlaps with the proliferation phases. Therefore, there is a need to develop different nanomaterials with outstanding anti-inflammatory properties for the immunoregulation of different types of chronic wounds and their treatment. In this context, the identification of markers that specifies the transition from the inflammatory phase to the proliferation phase using immunomodulatory nanomaterials would be a feasible strategy. Overall, nanotherapeutic-based strategies present promising approaches for the clinical treatment of chronic wounds by providing excellent antibacterial effects, reduced bacterial drug resistance, reducing the inflammatory phase, and shortening of the wound healing time for the promotion of wound healing.

**Table 1:** A list of recent nanotherapeutic approaches for wound healing applications along with wound type being treated, loaded drugs/growth factors, and findings.

Type of Nanomaterials	Wound Type	Drugs/Therapeutic Agents/Growth Factors	Findings	Reference
Poly (ethylene terephthalate) (PET) nanofibers	Acute (skin wound)	Anionic antibiotics piperacillin/tazobactam (PT)	High loading efficiency and sustained delivery for PT, reduced bacterial load	[116]
Poly (lactic-co-glycolic acid)/gelatin (PLGA)/gelatin nanofibers	Chronic (diabetic wound)	Liraglutide (Lira)	Shorter wound closure time, enhanced collagen deposition and alignment, increased blood vessel density	[117]
Poly (lactic-co-glycolic acid)-polyethylenimine nanoparticles	Acute (skin wound)	Nitric oxide (NO)	Strong bactericidal effect against methicillin-resistant *Staphylococcus aureus* (MRSA) bacteria, accelerated wound healing	[118]
α-gal nanoparticles	Chronic (diabetic wound)	-----------------------	Enhanced vascularization, re-epithelialization, granulation tissue formation, accelerated wound healing	[119]
Solid lipid nanoparticles	Chronic wound	Serpin A1 (A1) and host defense peptide LL37	Promotion of wound closure, reduction of bacterial contamination, and enhancement of anti-inflammatory activity	[120]
Liposome with silk fibroin hydrogels	Chronic (deep second-degree scald)	Basic fibroblast growth factor (bFGF)	Accelerated the wound closure, induced regeneration of vascular vessel	[121]
Photoluminescent gold nanodots	Acute (skin wound)	Antimicrobial peptide (surfactin; SFT), and 1-dodecanethiol (DT)	Enhanced antimicrobial properties and collagen deposition	[122]
Peptide dendrimers	Chronic (diabetic wound)	-----------------------	Smaller wound area percentage, improved wound healing	[123]
Fusidic acid nanoemulsion	Chronic (burn wound)	-----------------------	Reduction in bacterial load, wound contraction, and faster re-epithelialization	[124]
Recombinant human hair keratin nanoparticles	Acute (dermal wound)	-----------------------	Improved epithelialization, vascularization, along with collagen deposition and remodeling.	[125]
Chitosan nanoparticles	Chronic (prostatic wound)	Rebamipide	Improved re-epithelialization and faster wound healing	[126]
PLGA-liposome nanofibers	Acute (skin wound)	MicroRNA 145 (miR-145) and platelet-derived growth factor (PDGF)	Promotion of wound healing with enhanced vascularization and decreased wound size	[127]
Gelatin nanofibers	Chronic (burn wound)	anionic drug and hydrotalcite	Accelerated wound healing with strong antimicrobial activity	[128]
Silk fibroin nanoparticles	Chronic (ulcerative colitis)	Resveratrol	Reduced level of intracellular ROS, polarization of macrophages to type M2, restoration of damaged colonic epithelial barriers, reduced inflammatory reactions and level of intracellular ROS.	[129]
Poly (l-lactic acid) (PLLA) nanofibers	Chronic (diabetic wound)	Silica nanoparticles and dimethyloxalylglycine	Improved neo-vascularization and re-epithelialization with enhanced collagen deposition	[130]
Poly-(1,4-phenyleneacetone dimethylene thioketal)	Acute (full-thickness skin defect)	Stromal cell-derived factor-1α(SDF-1α)	Induction of wound vascularization, accelerated wound healing	[131]
Elastic liposomes with hyaluronic acid	Chronic (diabetic wound)	Epidermal growth factor (EGF), platelet-derived growth factor-A (PDGF-A), and insulin-like growth factor-I (IGF-I)	Reduction of wound size, improved skin permeation, and healing	[132]
Chitosan capped silver nanoparticles	Chronic (burn wound)	-----------------------	Shortening of the length of repair phases, enhanced re-epithelialization	[133]
Polyvinyl alcohol nanogels	Acute (skin wound)	Cerium oxide nanoparticles	Antimicrobial activity and rapid healing	[111]
Copper nanoparticles	Chronic wound	-----------------------	Increased vascularization, accelerated healing process	[134]
Chitosan hydrogels	Chronic (diabetic wound)	Silver nanoparticles	Promotion of antibacterial activity, enhanced healing	[135]
Polymeric composite dressings	Chronic (diabetic wound)	Calcium	Stimulated angiogenesis, collagen synthesis, accelerated wound healing	[136]
Fibrin nanoparticles	Acute (dermal wound)	Keratinocyte growth factor	Better cell proliferation and migration along with enhanced wound healing	[137]
Chitosan/Collagen blended nanofibers	Acute (full thickness skin wound)	Curcumin	Reduction in wound coverage area, improved healing	[138]
Collagen mats	Chronic wound	Inorganic polyphosphate (polyP)	Reduction in wound area, accelerated re-epithelialization rate and healing	[139]

### 5.2. Stem Cell Therapy-Based Strategies

In recent years, regenerative medicine has emerged as a revolutionary field to provide alternative therapeutic strategies to restore normal skin architecture and improve wound healing [140,141]. Stem cell-based therapy regenerative medicine for wound healing and skin regeneration has garnered much interest owing to its properties viz., long-term self-renewal capacity, and differentiation potential to multiple cell types [142]. Among cell therapies for wound healing, stem cells and progenitor cells have received much attention and remaining stem cells that are close to wounds site heal the wounds due to the stem cell’s plasticity. Moreover, stem cell-based therapy has provided great potential for healing chronic wounds which otherwise cannot be healed using conventional therapies. The stem cell-based therapy for chronic wound healing utilizes various processes such as interactions and actions of growth factors, the regulation of inflammatory processes, and the stimulation of immune processes for accelerating the vascularization and re-epithelialization [143]. A number of clinical and preclinical trials using stem cells in recent decades have presented profound impacts on quality wound healing [18,144]. The therapeutic potential of stem cell-based wound therapy is largely attributed to its ability to secrete pro-regenerative cytokines and growth factors for the promotion of skin regeneration during the treatment of chronic wounds [141]. In addition, autologous stem cells have excellent differentiation potential, support angiogenesis, and are well-tolerated by the patient with minimum adverse reactions. The tremendous ability of stem cells to transform into any other types of cells makes them a perfect choice to support the natural healing process to promote cell proliferation and accelerate wound closure. Earlier reports demonstrated the beneficial role of stem cells in wound healing as they directly and indirectly stimulate the residing cells in skin tissue, release active biomolecules, modulate inflammation, and remodel the ECM [145]. Owing to the presence of these beneficial effects, one can anticipate accelerated wound healing with a prevention of wound contraction and scar formation, expeditious wound closure, and regeneration with administration of stem cells. Nevertheless, the main clinical focus of stem cell-based therapy is to target improved quality wound healing for wound care.

Among the different types of stem cells, adult mesenchymal stem cells (MSCs), embryonic stem cells (ESCs), and the more recently explored induced pluripotent stem cells (iPSCs) present the main sources of stem cells that are utilized for wound healing and skin regeneration [146]. ECSs as a stem cell source were minimally utilized for wound healing due to the associated ethical concerns. A representative list of the different sources of stem cells that are utilized in wound healing is shown in Table 2. MSCs remain most convincing source of stem cells for wound healing owing to its ability to modulate inflammation, enhanced angiogenesis and granulation tissue formation, antimicrobial effects, reduction of scars, and promotion of fibroblasts [147,148]. MSCs are obtained from a plethora of sources such as bone marrow, adipose tissue, umbilical cord blood, Wharton’s jelly stem cells, and amniotic fluid. MSCs are involved in all the four phases of wound healing for the promotion of wound healing by facilitating migration to the wound site and the stimulation of angiogenesis, growth factors/cytokines release, and re-epithelialization. Bone marrow-derived mesenchymal stem cells were utilized for the first ever human study for the treatment of severe skin burns injuries followed by skin grafting and reported improved neo-vascularization and relief from the pain [149]. In another similar study with burn wounds, patient’s own bone marrow stem cells (BMSCs) were transplanted to the wound surface and treated burn patients involving 80% total body surface area (TBSA) and hypertrophic scarring followed by covering with acellular dermis support matrix [150]. The transplantation of stem cells resulted in reduced wound contraction, modulation of ECM, and enhanced angiogenesis. Furthermore, another clinical study was performed using biodegradable collagen membrane (Coladerm) and autologous bone marrow-derived MSCs and skin fibroblasts for the treatment of chronic non-healing wounds (diabetic ulcer). The results revealed a decrease in the wound size and enhanced vascularization after 29 days of combination treatment [151].

In a recent study, the transplantation of MSCs reported improved cutaneous wound healing through paracrine signaling of vascular endothelial growth factor that was released by MSCs [164]. Despite the promising role of MSCs in cell-therapy-based approaches for wound care, there remains a few limitations such as long-term safety and poor viability after implantation. In order to improve the survival of transplanted MSCs after implantation, various strategies have been employed [165]. One of the strategies includes scaffold-based therapeutic strategies along with stem cells. Herein, stem cells to be transplanted were first seeded on a three-dimensional scaffold matrix that was derived either from natural or synthetic polymers followed by delivery to the wound site after transplantation. This approach greatly improved the stem cell survival, preservation of cellular functions, and wound healing using stem cells and decellularized silk fibroin scaffold [166,167]. Kamolz et al. utilized Matrigel with Matriderm for the delivery of MSCs to the wound site [168]. Furthermore, the expansion of this approach incorporated growth factors within the scaffolds to improve the vascularization and wound repair [169]. These reports demonstrate the better wound healing effect using stem cells and scaffold-based delivery systems with the addition of growth factors. Furthermore, several compelling clinical studies using stem cells have been conducted with scaffold-based delivery systems for the acceleration of wound healing in chronic wounds [141,170]. However, only a few studies involving natural and synthetic biocompatible scaffolds and stem cells are FDA-approved. Out of 90 clinical trials that were registered for the evaluation of stem cells healing potential, only 23 trials utilized biomaterial scaffolds for stem cell delivery to the wound site and healing chronic wounds as accessed from www.clinicaltrials.gov (US National Institutes of Health (NIH) (accessed on 20 May 2022). Another strategy to improve stem cell survival rate at the wound site includes genetic modification of the stem cells. In a recent study, BM-MSCs overexpressing TGF-β3 improved the wound healing process with reduced scar formation [21]. In another study, epidermal stem cell overexpressing Cd271 reported increased cell differentiation, proliferation, and migration in a burn wound model [171]. Furthermore, the orientation of MSCs play a vital role in improving wound healing and optimal tissue regeneration. Utilizing this strategy, 3D printed polylactic acid (PLA) that was coated with nanotubes of Halloysite (aluminosilicate clay mineral) demonstrated beneficial effects of MSCs orientation on wound healing [172]. In another study, 3D-printed polycaprolactone scaffold that was coated with nanocellulose and seeded with MSCs revealed the promotion of cellular growth and differentiation for the wound healing process [173]. In recent years, MSCs-derived exosomes, which is source of various growth factors and cytokines proved to be a promising newer stem cell-based strategy for accelerating the wound healing process by promoting cell migration and proliferation, angiogenesis, re-epithelialization, and activating several signaling pathways (such as Wnt/β-catenin, AKT, ERK, and STAT3) [169,174]. Adipose-derived MSCs are also very commonly utilized in wound healing applications due to its high accessibility, minimal invasiveness, and lack of ethical limitations [156,175]. Recently, exosomes that were derived from adipose-derived MSCs have shown accelerated wound healing with a significant increase in the wound closure rate by attenuating the inflammation phase [176,177]. Other MSCs source, umbilical cord blood stem cells (UC-MSC) showed promising therapeutic effects in the treatment of diabetic chronic wounds through higher cell proliferation and the deposition of collagen [178]. Although clinical trials with UC-MSC-based therapy are less, these stem cells present advantages over bone marrow MSCs in terms of high numbers of cells isolation, high cell yield, and senescence retardation [179]. In a recent study, a combination of MSCs with natural substances have demonstrated enhanced healing properties using a combination of human umbilical cord-derived MSCs and platelet-rich plasma cryogels in third degree burn wounds with significantly increased angiogenesis, wound closure, and re-epithelialization compared to controls [180]. Platelet-rich plasma is derived from blood and is rich in growth factors and antimicrobial properties that are a prerequisite for the improvement of wound healing. Another stem cells source, human Wharton’s jelly stem cells (HWJSC), also reported better healing in burn wounds when transplanted with acellular dermal matrix [181]. 

Apart from these MSCs sources, there are various other stem cells that are harbored by skin that accelerate wound healing. The epidermis harbors different stem cells such as bulge area stem cells, interfollicular stem cells, and sebaceous gland stem cells [182]. On the other hand, the dermis contains two types of stem cell populations viz., hair follicle stem cells in dermal papilla and perivascular stem cells. These stem cells are reported to accelerate the wound healing process with minimum ethical concerns and immune rejections [158]. However, in order to have more substantial understanding regarding their usefulness in wound healing, the exploration of more clinical trials is required in humans to confirm the effects.

In order to overcome the shortcomings of MSCs and ESCs, a new stem cells source, induced pluripotent stem cells (iPSCs) has been unleashed in the regenerative medicine domain [125,141,183]. iPSCs are remarkably similar in morphology with ESCs but without any ethical concerns and potential for immunological rejection that is associated with ESCs [184]. iPSCs are reprogrammed cells that are derived from somatic cells combination of reprogramming factors (e.g., Sox2, Klf4, Oct3/4, and c-Myc) and are considered as a rising star in regenerative medicine and opened unprecedented opportunities for transplant therapy. Bilousova et al. demonstrated differentiation of iPSCs into epidermis, sebaceous glands, and hair follicles [185]. In another study, epithelial stem cells that were derived from iPSC (iPSC–EpSC) indicated reconstitution of hair follicles [186]. Furthermore, various research studies have demonstrated either iPSCs or iPSCs-derived exosomes for the facilitation of wound healing with enhanced wound closure, collagen synthesis, and angiogenesis [162,187,188]. In recent studies, iPSCs from different somatic cells such as fibroblasts, keratinocytes, and folliculogenic human epithelial stem cells showed significant progress in bioengineering of skin substitutes [146]. Furthermore, Yan et al. utilized iPSCs- derived micro-vesicles containing microRNA (miR-16-5p) for enhanced re-epithelialization and collagen deposition in burn wounds [163]. The iPSCs-based stem cells strategy can be combined with genome editing tools for permanent corrective therapy for chronic injuries using clustered, regularly interspaced, short palindromic repeats (CRISPR), transcription activator-like nuclease effector (TALEN), and zinc nuclease finger (ZFN) tools. Overall, stem cell-based therapy and different strategies provided an upper hand over conventional approaches for enhanced wound healing. However, the exploration of stem cells sources, processing methods, and administration routes should be more studied in order to realize the real clinical situation in regenerative wound healing.

### 5.3. D Bioprinting-Based Strategies 

**Figure 4:**
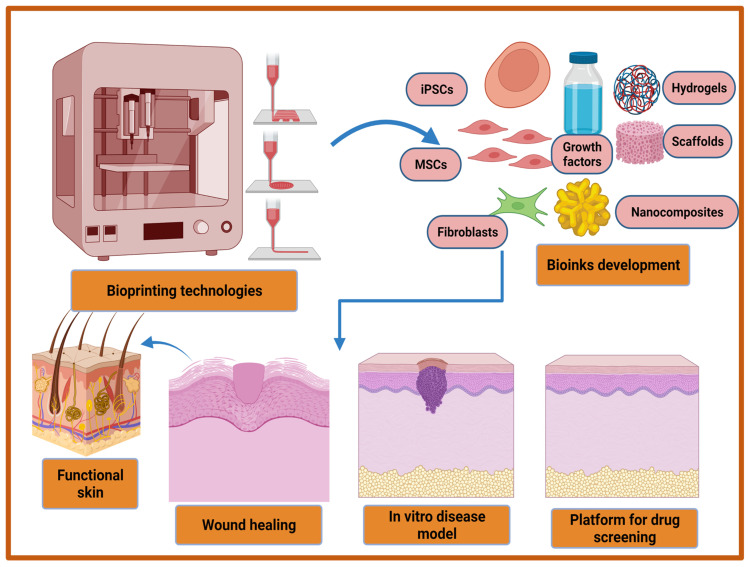
Recent advancements in 3D bioprinting technologies and bio-inks development for improved wound healing, in vitro disease model development, and the fabrication of high throughput platform for drug screening.

In last two decades, several therapeutic modalities have been extensively utilized for the treatment of acute and chronic wounds. However, most of the treatment options were manual and their success was hindered by the requirement of a relatively long time to cover large wounds or burns. To overcome this limitation, 3D bioprinting has emerged as a rapid and high throughput automation technology in recent decades to address these challenges in regenerative medicine, including wound healing. The three-dimensional (3D) bioprinting technique is an additive manufacturing technique that provides a promising strategy for the fabrication of biocompatible artificial skins through a precise layer by layer deposition of living cells, biomaterials, biomolecules, and growth factors. This automated technology is a flexible tool which is superior for clinical uses in terms of accuracy and functionality. The advantages of 3D bioprinted skin constructs for wound healing and skin regeneration include (1) automation and faster fabrication which lesser time and cost; (2) flexibility to introduce different cells and biomolecules during the process for the promotion of innervation, pigmentation, and vascularization; (3) the ability to precisely deposit multiple biomaterials and cells in different positions; and (4) large-scale fabrication with good plasticity and extensibility [189,190,191]. The bioprinted skin equivalents closely mimic the native skin architecture and heterogenicity via the precise deposition of multiple cells and biomaterials [192]. The bioprinted skin constructs should fulfill various features in terms of functional and compositional properties. Firstly, bioprinted skin substitutes should be able to transport nutrients and wound exudates. Secondly, bioprinted equivalents should be able to accurately deposit different skin cells such as keratinocytes, fibroblasts, adipocytes, melanocytes, Langerhans cells, etc., at particular layers and locations. Thirdly, porosity, degradation, and mechanical properties should closely mimic the native skin structure, and finally, the bioprinted structure should be robust, biocompatible, biodegradable, and should be able to sustain external forces and pressures that are present in in vivo conditions [193,194]. Furthermore, with the recent advancement in 3D bioprinting technologies it may be utilized for the development of an in vitro 3D disease model, and high throughput platform for drug screening (Figure 4). Moreover, a multi-thronged approach using application-specific bioprinting systems is required to develop personalized therapies for different types of wounds using autologous cells.

There are mainly four different types of bioprinting technologies that are being utilized for cutaneous wound healing and skin tissue regeneration. The different types of bioprinting techniques based on their native prototypes include extrusion-based, droplet-on-demand (inkjet)-based, laser-based, and stereolithography-based bioprinting technologies [195,196,197,198]. Among all these technologies, extrusion-based bioprinting (EBB) techniques are the most common for skin bioprinting owing to different advantageous features such as high printing speed, cost-effectiveness, accessibility, capability to replicate complex tissues, and the ability to print wide range of viscous biomaterials [199]. In EBB, to deposit the predesigned geometrically well-defined 3D complex structures of skin, the extrusion of continuous stands of bio-inks through a nozzle is generally powered by pneumatic pressure, screw-based, piston, or microfluidic mechanisms [200]. However, the EBB technique suffers from one limitation that is frequent clogging problems with different types of nozzles. Similarly, other abovementioned bioprinting techniques also have various pros and cons that need improvement in terms of bioprinting device designs and methodologies. In order to overcome the limitations of bioprinting technologies, newer and/or improved bioprinting technologies and the utilization of hybrid bioprinting systems combining two different existing bioprinting systems would be prospective strategies for skin bioprinting. The examples of new and improved forms of existing bioprinting technologies includes continuous liquid interface production (CLIP) [201], multi-material multi-nozzle 3D printing (MM3D) [202], and computed axial lithography (CAL) [203]. CLIP-based bioprinting is better than conventional stereolithography (SLA) printing methods as it has 100 times faster printing speed and creates a ‘no polymerization zone’ using an oxygen-permeable window below the UV projection to ensure continuous SLA process [201]. CLIP can be extended for printing multi-layer skin constructs on a larger scale using different photo-initiators, visible light-based techniques, and biocompatible photopolymers. MM3D-based bioprinting employs a voxel-by-voxel approach instead of the conventional layer-by-layer approach using a specialized printhead with high frequency and ability to switch between up to eight different materials [202]. This bioprinting approach provides benefits to skin tissue printing as it allows the maintenance of stiffness difference, not only between the different layers but also within length and breadth of any single layer. This method of bioprinting and CAL was developed in 2019 much later than CLIP in 2015 for skin bioprinting applications. Therefore, much exploration is still required using this bioprinting technology for skin tissue regeneration. The other revolutionary bioprinting method includes CAL, which utilizes volumetric construction instead of conventional layer-by-layer construction for 3D bioprinting [203]. The advantages of CAL include the utilization of visible light instead of UV light or laser light, and printing flexibility over a preexisting solid object. Besides the new or improved bioprinting techniques, another approach to improve skin bioprinting includes the utilization of hybrid bioprinting systems, which involves two or more existing bioprinting techniques. For example, Kim et al. have demonstrated the construction of epidermal and dermal skin layers by using inkjet and extrusion bioprinting techniques, respectively [204]. Moreover, hybrid bioprinting-based approaches hold great potential for skin bioprinting and needs further exploration in order to realize its full potential. 

The other important criteria for successful wound healing and skin engineering using 3D bioprinting techniques include biomaterial ink or bio-ink [205,206]. Bio-inks are a combination of biomaterials, cells, and growth factors/biomolecules, while biomaterial ink comprises of everything that is present in bio-ink but cells. A wide range of biomaterial hydrogels are being employed for skin bioprinting viz., chitosan, gelatin, collagen, silk fibroin, alginate, cellulose, hyaluronic acid etc., along with synthetic biopolymers to improve the mechanical properties of bioprinted constructs [207,208]. Whether natural or synthetic biopolymers are used as bio-inks, there are certain crucial properties that should be possessed by all bio-inks. These properties include biocompatibility, high availability, biodegradability, good printing ability, mechanical stability, and the ability to maintain high shape fidelity after the bioprinting process. In addition, bio-inks utilize living cells which can impact the immune response after implantation, therefore, the selection of cells should be considered keeping in mind this crucial point. In skin bioprinting, either primary skin cells such as fibroblasts, melanocytes, keratinocytes, or stem cells are preferred for the fabrication of skin constructs while maintaining all cellular activities after bioprinting [209]. Another important property of bio-ink includes viscoelastic behavior that influences the skin bioprintability and also affects the cell migration, proliferation, and remodeling of the ECM [210]. Regardless of the bio-inks or bioprinting techniques that are utilized, skin bioprinting for wound healing applications is carried out using two strategies, namely ex vivo and in situ bioprinting. In ex vivo bioprinting, an extrusion-, inkjet-, or laser- based bioprinting method is utilized for printing epidermal and dermal layers of the skin followed by in vitro maturation, if necessary. After maturation, the bioprinted skin constructs are grafted to the wounds of the patient. On the other hand, bio-inks (containing skin cells in biomaterial hydrogel) are directly bioprinted on the injured site in an in situ bioprinting approach using the most common handheld extrusion bioprinter to reproduce the skin structure followed by bio-inks crosslinking, if required [211].

Lee et al. reported the first bioprinted skin in 2009 using collagen hydrogel and human dermal fibroblast-based bio-ink [212]. Furthermore, Koch et al. fabricated skin equivalents by utilizing a collagen hydrogel-based bio-ink containing keratinocytes and fibroblasts [213]. At the same time, Binder et al. employed a 3D inkjet-bioprinter using human fibroblasts and keratinocytes for fabricating skin substitutes for wound healing [214]. Afterwards, significant progress was reported in the skin bioprinting filed using a wide range of bio-inks and bioprinting techniques. 3D bioprinted skin equivalents serve as an alternative to traditional skin grafts for the regeneration of skin tissue structure with appendages and overcome the problem of donor and surgery requirements [189,215]. Moreover, this emerging skin bioprinting technology has been applied for the treatment of various chronic and non-healing wounds such as burn wounds, pressure ulcers, venous ulcers, and diabetic foot ulcers. In a study, a hand-held 3D bioprinting instrument was developed for the treatment of full thickness burn wounds in porcine models with reduced scar formation [216]. This hand-held bioprinter allows in situ bioprinting of sheets of skin tissue, maintaining the cells heterogenicity and compositional variations in different skin layers. This system utilizes epidermal and dermal skin cells and different biopolymers such as collagen, alginate, and hyaluronic acid hydrogels for skin bioprinting. In another study, full-thickness human skin equivalent with structural and biomechanical similarity to native skin were fabricated along with an undulated pattern of the unique dermal-epidermal junction [217]. In the last decade, tremendous progress has been witnessed in the skin bioprinting field [217,218,219]. Using 3D bioprinting technologies, initially only dermis printing [220] was attained followed by bilayered dermal and epidermal layer bioprinting [217,221,222], and skin trilayers bioprinting (epidermis, dermis, and hypodermis) [223,224]. Table 3 summarizes some of the most important studies using 3D bioprinting technology and constructs for accelerating wound healing and skin regeneration. The tremendous progress in skin bioprinting is attributed to exploration of newer and improved bio-ink formulations, availability of a wide range of bioprinters with different capabilities and properties, tremendous progress in bioprinting technologies, and the escalation of cell biology and metabolism knowledge before, during, and after the bioprinting process.

In a new strategy for skin bioprinting, pro-angiogenic peptides were incorporated in hydrogel patches for the fabrication of 3D-bioprinted skin constructs. These improved bio-inks exhibited excellent printability and vascularization properties and provided beneficial roles in skin wound healing [231,232]. In a recent study, an angiogenic 3D-bioprinted peptide patch was developed for the improvement of skin wound healing. Herein, biocompatible biopolymers gelatin methacryloyl (GelMA) and hyaluronic acid methacryloyl (HAMA) that were covalently conjugated with QHREDGS peptide were utilized for bioprinting. The bioprinted peptide-containing patch demonstrated improved angiogenesis, biocompatibility, and wound repair both in in vitro and in vivo conditions [233]. In another study, a bioprinted tissue co-culture platform using a mixture of plasma-derived fibrinogen-containing factor XIII, fibronectin, thrombin, and macrophages (an FPM “bio-ink”) was utilized for stimulated wound closure and re-epithelialization in murine dermal wound model through extrusion bioprinting [226]. Until now, a bioprinted skin equivalent containing all skin elements has not been available, but this emerging field is still in the developing stage and require more exploration in bioprinting technologies and bio-inks for skin regeneration and wound healing. 

### 5.4. Extracellular Matrix (ECM)-Based Strategies 

**Figure 5:**
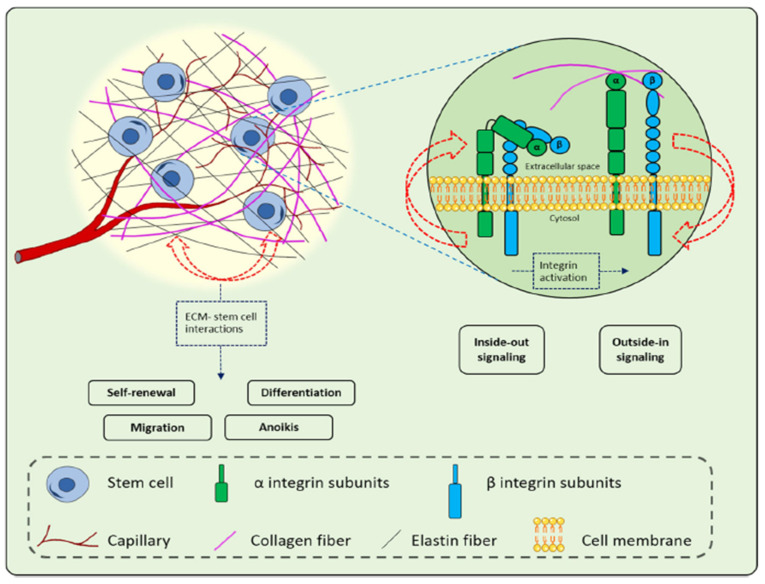
Interactions of the ECM fibers and stem cell integrins through inside-out signaling and outside-in signaling for the modulation of cell migration, proliferation, and differentiation during the wound healing process. Inside-out signaling allows integrins to interact with ECM fibers for integrin activation, while outside-in signaling produces the desired changes in stem cells. Reproduced from ref. [240] with permission from Elsevier.

During wound healing, the matrix of the dermal layer generally directs all the phases of healing process after skin damage. However, in the case of chronic wounds, healing is stalled due to a lack of functional ECM in the dermal matrix, which is responsible for stimulating healing with the aid of external factors [234]. The presence of highly activated matrix metalloproteinases, increased abundance of senescent fibroblasts, and a persistent inflammatory phase in chronic wounds degrades the ECM which causes poor wound healing [235]. The restoration of functional ECM in chronic wounds stimulates, directs, and systematizes the healing process for facilitating wound closure. Therefore, in order to improve the healing process in chronic wounds, a proper understanding of ECM components and its interactions with cells is a prerequisite. The ECM is an extensive three-dimensional molecular network, which provides structural integrity and mechanical support to different tissues, including skin. The ECM structure comprises of a variety of macromolecules such as fibrous proteins (collagen, fibronectin, vitronectin, elastin, and laminin), glycosaminoglycans, and proteoglycans [236]. The ECM plays a crucial role in directing the wound healing process by managing cell behavior (adhesion, migration, proliferation, and differentiation) and cellular activities in the damaged skin for tissue repair and regeneration [237,238,239]. In addition, the dynamic ECM structure acts as a reservoir for the storage and delivery of different growth factors and cytokines to provide biological signals. The ECM composition undergoes changes during the wound healing phases and facilitates active interactions of cells and growth factors bioavailability to accelerate the wound healing process [240]. In the hemostasis phase, provisional ECM that is made up of fibrin and fibronectin is formed to support cell adhesion and migration. Furthermore, the ECM provides a 3D structural framework during the inflammation phase for the clearance of microorganisms. Thereafter, provisional ECM is formed during the hemostasis phase disrupts and is replaced with the formation of granulation tissue (fibronectin) to support collagen deposition. Finally, collagen I is replaced with the synthesis of collagen III during remodeling to provide mechanical support. In skin tissue, ECM components communicate with precursor cells and stem cells that are present in skin tissue through integrin and non-integrin receptors during the repair process [240]. The interactions of ECM and stem cell integrins play crucial roles in the wound healing process by modulating various cellular key events (Figure 5). The key events of wound healing such as migration, proliferation, differentiation, and cellular death are mediated through outside-in signaling of integrins. As a result, if the ECM components and stiffness dysregulates, the reciprocal interactions between the cells and ECM and tissue regeneration are ultimately disrupted leading to pathological conditions. Therefore, a better understanding of the ECM components and their roles in the wound healing process may improve the strategies that are related to wound care. 

Although the ECM-based approach for the stimulation of wound healing is not very new, how the different ECM components provide a ‘jump-start’ to the healing process are still not much explored. In recent decades, various ECM-based strategies have been employed for the treatment of chronic wounds. The most prominent strategy includes the utilization of bioengineered tissue-derived or synthetic ECM scaffolds to promote wound healing. The ECM-based scaffolds mediate the recruitment of cells, delivers growth factors to injured wound site, and provides mechanical support. There are different types of ECM-based scaffolds viz., cellular-derived ECM [237], synthetic biomaterials-based scaffolds/hydrogels [241,242], and decellularized ECM (dECM) scaffolds [243,244]. Among all the ECM-based approaches, the dECM-based approach presents the most promising approach in recent years as alternative emerging strategies to overcome the hurdles that are associated with the organ/tissue transplantation viz., donor shortage and disease transmission risk. dECM that are either derived from tissues or cells are devoid of cellular components and serves as reservoirs for cell-matrix interactions and site-specific bioactive components, offering various advantages for the regeneration of tissues. The preserved non-immunogenic ECM mimics native three-dimensional architecture and non-immune environment for the repair and regeneration of skin tissues [234]. The skin repair is mediated through the utilization of dECM scaffolds as they retain the porous bilayer structural architecture of skin along with bioactive molecules, elasticity, and adhesiveness of the skin and vessel structure [245,246]. In addition, dECM degradation products do not illicit any toxicity while skin is regenerated and, in fact, promoted the formation of tube-like structures for skin regeneration [247]. In a study, dECM-based scaffolds facilitated the adhesion of keratinocytes due to maintained basement membrane. Additionally, dECM with preserved dermal structure (papillary and reticular dermis) is reported to promote the growth of vascular cells [248,249]. In another study, Choi et al., demonstrated a reduction of wound contraction and less scar formation due to the release of basic fibroblast growth factors from dECM [250]. Furthermore, Brouki Milan et al. demonstrated enhanced cutaneous wound healing as revealed from reduced collagen deposition, faster re-epithelialization, and accelerated wound closure rate using a decellularized dermal matrix (DDM) scaffold reseeded with human umbilical cord perivascular cells (HUCPVCs) [251]. In another study, Groeber et al. reported the fabrication of a bilayared skin alternative that was suitable for skin grafting using decellularized porcine jejunum that was reseeded with dermal fibroblasts, human epidermal keratinocytes, and human dermal microvascular endothelial cells [252]. Owing to dECMs ability to preserve endogenous angiogenesis factor and vasculature, dECM scaffolds have been employed in diabetic pressure ulcers (DPU) treatment wherein high vascularization is required to promote healing [253]. In another study, accelerated wound healing in DPU’s has been reported by shortening the inflammation period, regulating granulation tissue formation, pro-angiogenesis activity, and epithelial regeneration using dECM scaffolds [251]. These regulatory activities that are mediated by dECM scaffolds may reduce the scar formation. In recent years, many acellular products such as AlloDerm^®^ regenerative tissue matrix and Oasis^®^ wound matrix has been commercialized for clinical applications and have shown promising results [254]. However, in order to become skin alternatives, ECM-based acellular products need thorough optimization of fabrication and characterization procedures. In order to attempt this, decellularized human placenta-derived ECM-based scaffolds successfully modulated healing in full thickness wounds along with hair follicle formation [250,255]. The reconstitution of hair follicles and sweat glands is the main criteria for native skin regeneration, which was not observed in earlier clinical products. Overall, decellularized ECM scaffolds and different types of ECM mimics presents great potential for chronic wound treatment and may augment wound healing many folds by incorporating growth factors and antibiotics.

### 5.5. Platelet-Rich Plasma (PRP)-Based Strategies

Platelet-rich plasma (PRP)-based endogenous therapeutic technology has the potential to stimulate and accelerate tissue healing including wound healing and has garnered much attention in recent years in regenerative medicine [256,257]. PRP is an autologous biological product containing higher amounts of platelets compared to circulating blood and thus represents an increased concentration of growth factors which is a prerequisite for wound healing [258]. PRP is also called an autologous platelet concentrate, autologous platelet gel, or plasma-rich growth factors and plays a pivotal role in wound healing as platelets have hemostatic function and promote skin cell proliferation and tissue expansion [259]. The growth factors pool in PRP includes platelet-derived growth factor (PDGF), epidermal growth factor (EGF), fibroblast growth factor (FGF), transforming growth factor beta 1 (TGF-β1), insulin growth factor-1 (IGF-1), keratinocyte growth factor (KGF), vascular epithelial growth factor (VEGF), etc., that promote cell migration, proliferation, and differentiation, for the initiation of wound healing. Growth factors plays pivotal roles in complex wound healing processes and skin tissue regeneration as they act as signaling molecules which influence the cellular metabolism [260]. Almost all cell types that are present in the skin are involved in producing growth factors and different cells produce different types of growth factors during the wound healing phases. Each growth factor demonstrates more than one effect and controls cellular processes such as cell migration and proliferation, ECM remodeling, and angiogenesis which provides an ideal environment and promotes the wound healing process. The paramount importance of growth factors in wound healing lies in the fact that a single growth factor is not sufficient for the treatment of hard-to-heal chronic wounds, especially diabetic foot ulcers [261]. The advantages of using PRP technology in wound healing include easy methodology, safety, and cost effectiveness [262]. PRP is obtained from patients’ blood followed by a simple centrifugation process for its preparation. Therefore, by controlling the centrifugation parameters and the protocol that is being used for the activation it is possible to control the dose of growth factors that are provided by PRP. PRP technology has more long-lasting effects than conventional therapies and it is safer to use as it is obtained from the same patient (autologous). Additionally, it has potential antibacterial activity besides the potential impact in the reduction of economic costs for standard treatment regimens. Moreover, PRP treatment-based therapy does not replace the conventional therapies but serves as an alternative treatment. 

In recent years, the usage of PRP technology in wound healing is steadily increasing in various preclinical and clinical studies. The beneficial role of PRP-based treatment in wound healing has been extensively reported in humans [263,264], horses [265], dogs [266], and other species [267,268]. The first clinical application of PRP was performed on chronic leg ulcers using collagen-embedded platelet proteins with the induction of vascularized connective tissue in wounds [269]. Thereafter, different forms of PRP such as gel, solution, or injection have been employed in different wounds with varying etiologies. The different wounds wherein PRP treatment has been reported in various animal and human trials include acute cutaneous wounds [270], chronic skin ulcers [271], burns [272], and plastic and cosmetic surgery [273]. Furthermore, PRP treatment did not elicit any adverse reactions in terms of risk of infection or hypersensitivity reactions during clinical trials [274]. Among the human clinical trials, the main use of PRP treatment is related to hard-to-heal chronic wounds wherein there is an impairment of the wound healing due to a low concentration of growth factors, persistent inflammation, imbalance of pro-inflammatory and anti-inflammatory cytokines, and excess reactive oxygen species [275]. PRP, being rich in different growth factors, have a strong regenerative capacity which shortens the wounds recovery time [262]. In a study, PRP-based dressing treatment accelerated wound healing in skin ulcers and promoted the proliferation and migration of fibroblasts and mesenchymal stem cells (MSC) with faster neovascularization in clinical patients [276]. In another clinical study with 150 patients, the topical application of PRP in diabetic foot ulcers resulted in early complete closure of wounds with the formation of healthy granulation tissue [277]. In other study, the combination of topical application of PRP gel and subcutaneous autologous PRP injections in non-healing wounds demonstrated a significant reduction in the wound size, reduction in pain and inflammation, and potential safety in all the treated patients without any side-effects [263]. Moreover, PRP treatment has successfully demonstrated faster wound healing in different acute and chronic wounds with different etiologies. However, to develop final PRP-based therapeutic product, more standardization and characterization of different PRP preparation methods would be required [278]. In addition, PRP adjunctive treatment has been combined with either cell therapy or skin grafts in various clinical trials and does not delineate the actual potential of only PRP treatment. Nonetheless, recent clinical studies demonstrated improved wound healing outcomes suggesting its therapeutic potential in healing different types of chronic wounds. 

### 5.6. Cold Atmospheric Plasma Therapy-Based Strategies

**Figure 6:**
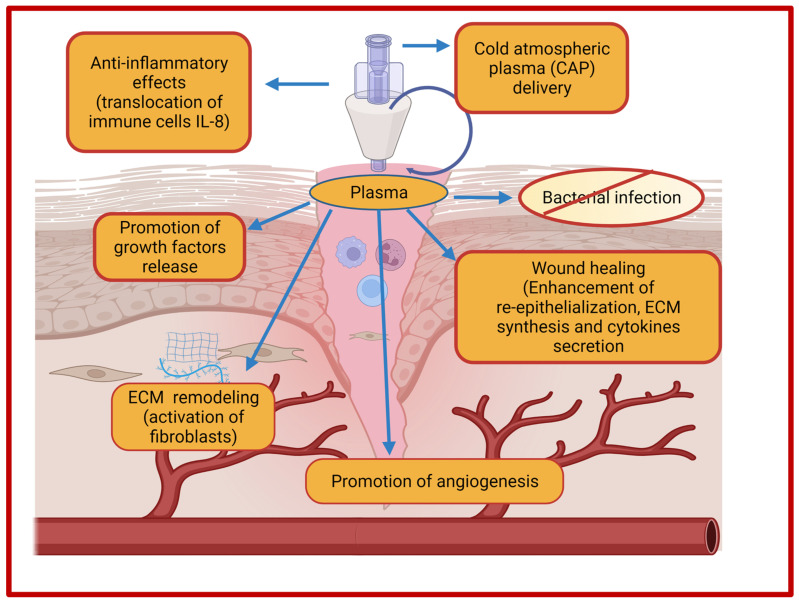
Effects of cold atmospheric plasma (CAP) on wound healing.

Plasma-based treatment strategies have been employed in biomedical engineering for the last 20 years in ulceration and cancer therapy. Plasma represents a fourth state of matter and is classified into thermal and non-thermal (cold) plasma. In thermal plasma, all the particles (electrons and heavy particles) are in thermal equilibrium, while non-thermal or cold plasma contain particles which are not in thermal equilibrium. In recent years, plasma-based therapy for wound healing has gained much attention owing to its beneficial properties. Cold plasmas, also termed as non-thermal plasma, gas plasma, or physical plasma, are most common in biomedical research as it utilizes a lower temperature (<40 °C) which is compatible for biomedical applications. Cold plasma is an ionized gas near room temperature that is comprised of charged particles (electrons, diverse reactive oxygen, and nitrogen species) and neutral particles (neutral atoms and molecules) [279]. In addition, the plasma cocktail contains UV irradiation and an electric filed that in combination with different ions and reactive species mediates biological effects that are necessary for tissue regeneration. The generation of plasma occurs due to a bombardment of electrons and photons with sufficient energy with the neutral atoms and molecules that are present in the gas phase. There are two primary sources for discharge of low-temperature plasma viz., dielectric barrier discharge (DBD) and non-DBD type atmospheric pressure plasma jet (APPJ) [280]. Moreover, cold atmospheric plasma (CAP) acts as an innovative intervention as it employs multimodal mechanisms of action for the treatment of chronic wounds and promotion of wound healing [281]. Cold plasma has the potential to reduce the bacterial load and induce the hemostatic process of wound healing due to the generation of the anti-infective reactive oxygen and nitrogen species. Additionally, cold plasma treatment renders the inactivation of microbial species through the generation of reactive oxygen species (ROS) and nitrogen species (RNS) and demonstrated potential in tissue regeneration in case of wounds. Among reactive nitrogen species, nitric oxide (NO) is most common for bacterial load reduction, promotion of ECM remodeling, and angiogenesis [282]. ROS and NO together mediate the enhanced expression of growth factors in wound regions and ultimately regulate the wound contraction and re-epithelialization [283]. The effects of cold atmospheric plasma on the improvement of wound healing are depicted in Figure 6.

The cold plasma utilizes both physical and biological mechanisms for wound healing. The physical mechanism of plasma-based treatment involves the generation of free radicals and reactive species for the desired wound healing-promoting effects, while the biological mechanism exploits various cellular processes that are responsible for DNA and cell membrane damage of bacteria. The advantages of using cold plasma in wound healing includes simplicity in use, non-invasive nature, wound acidification, antimicrobial effects, stimulation of cellular proliferations, enhanced microcirculation, and ultimately reduced treatment costs [279,284,285,286]. Wound acidification promotes the wound healing process due to decreases of pH in the aqueous medium that is induced by cold helium plasma treatment [285]. Currently, there are three certified and approved cold plasma devices for biomedical uses. The first plasma device was certified as a medical device Class IIa in 2013 under the name of kINPen^®^MED by INP Greifswald/neoplas tools GmbH, Greifswald, Germany) after evaluation in preclinical, clinical, and biological studies. kINPen^®^MED is a pen-sized atmospheric plasma jet (APPJ) device that was initially developed for laboratory usage but later scaled up also for commercial purposes. The intended use of kINPen^®^MED included the treatment of acute wounds, infected wounds, pathogen-induced skin diseases, and chronic wounds [287]. Moreover, kINPen MED device represents the largest and best elaborated prospective for diabetic foot ulcers (chronic wounds). Thereafter, a DBD source-based plasma device PlasmaDerm^®^ VU-2010 was developed by CINOGY Technologies GmbH, Germany and CE-certified by MEDCERT under ISO 13485. Consequently, the medical device SteriPlas was developed by Adtec Ltd., London, UK for the reduction of bacterial load and the treatment of acute and chronic wounds [288].

The effect of cold atmospheric plasma treatment on wound healing has been assessed in several in several clinical studies and case reports. The outcomes include the reduction of bacterial load, rapid healing, and faster migration of cells indicating the benefits of the cold plasma treatment in wound care. The ability to generate reactive species such as ROS and RNS for the induction of tissue regeneration provides additional benefits. Several preclinical and clinical studies in humans have been conducted for the treatment of chronic wounds without inducing any substantial side effects on normal tissue [286,289,290,291,292]. CAP treatment facilitated the transformation of chronic wounds to not become stagnating wounds through the modulation of inflammation, tissue-reactive species interactions, and the stimulation of different growth factors [285,293,294]. Fathollah et al. demonstrated accelerated wound healing with increased release of transforming growth factor (TGF)-β1 in diabetic wounds [290]. The researchers found stimulated cell proliferation, neovascularization, and the formation of the epidermis layer after cold plasma treatment. A representative list of the effects of CAP treatment on acute and chronic wounds healing is presented in Table 4 along with the device that was utilized, exposure time, and outcomes. Nevertheless, cold plasma treatment for the prevention of infectious skin diseases and enhanced wound healing holds immense scope for the future. In this context, plasma therapy expanded our knowledge in the domain of enhanced treatment options for wound healing.

### 5.7. MicroRNA (miR)-Based Strategy for Wound Healing

Chronic wound management and treatment is generally influenced by many multifactorial elements. To attempt this, a variety of treatment approaches have been employed for chronic wound healing. However, the currently employed treatment modalities are largely inadequate for chronic wounds. In recent years, a microRNA (miR)-based therapeutic approach involving either miR replacement or inhibitory therapy has garnered much attention for chronic wound healing as an alternative emerging mode of treatment [303]. miRs are short non-coding RNA molecules (~18–25 nucleotides in length) which are involved in various pathological and physiological processes viz., differentiation, metabolism, and development including wound healing process as it regulates the expression of mRNAs [304]. Earlier reports demonstrate the pivotal role of miRs in skin metabolism as it regulates the self-renewal and differentiation of epidermal skin stem cells [305]. Some of the highly expressed miRs in the skin epidermis includes miR-16, miR-152, miR-143, miR-205, miR-27b, miR-34a, miR-30b, miR-21, miR-27a, miR-125b, miR-125a, miR-126, miR-191, miR-200 family (-200a, -200b, -200c, -141, -429), miR-214, and miR-203 [306]. Earlier reports demonstrated the upregulation and downregulation of miR expression during the overlapping phases of wound healing [303]. In addition, during chronic wound healing, miRs expression is dysregulated which was otherwise expressed during the normal wound healing process. In an earlier study, the upregulation of 18 miRs and downregulation of 65 miRs was observed in diabetic wounds compared to non-diabetic rodents (control wound healing group) suggesting miRs as a therapeutic target for chronic wounds healing [307]. miR-based therapy presents a revolutionary advanced therapy for chronic wounds owing to miRs ability to regulate a group of genes using single miR. miR-based therapy for wound care relies on the ability of therapeutic miRs to upregulate the levels of beneficial miRs and downregulate the levels of damaging miRs through different methods. The upregulation of beneficial miRs can be achieved by using miR mimics (synthetic double stranded oligonucleotides). On the other hand, the downregulation of damaging miRs can be carried out using competitive inhibitors antagomiRs (complementary oligonucleotides). The success of mimics or antagomiRs delivery to the target miRNA depends on the binding affinity and resistance to destruction in different tissues [308]. Moreover, the identification and exploration of appropriate miRs that are involved in different phases of wound healing may provide effective third-generation genetic therapy for chronic wound healing through the regulation of multiple signaling pathways. However, in order to overcome intracellular and extracellular barriers, an appropriate design of delivery system is pertinent to ensure miR success as a gene therapy.

The role of miRs as regulatory molecules is observed in all phases of the wound healing process including the inflammation phase and proliferation phase involving angiogenesis, re-epithelialization, and granulation tissue formation to accelerate wound healing process [309,310]. A list of miRs and their role in different phases of wound healing is listed in Table 5. 

Furthermore, the importance of miRs in the regulation of wound healing phases was confirmed by delayed wound healing with a loss of a critical regulator that is responsible for the miR maturation (enzyme Dicer) [324]. In recent years, several miRs have emerged as wound healing regulators through recognition and binding with target genes to promote wound healing in chronic wounds. miR-132 promoted the enhancement of inflammation to the proliferation phase transition phase [325]. miR-31 promotes wound healing through the enhancement of proliferation and migration of keratinocytes [320]. In an earlier study, miR-27b accelerated wound healing in diabetic wounds [314]. In another study, wound contraction and collagen deposition regulation was mediated by miR-2 1 for the improvement of the wound healing process [326]. Subsequently, umbilical cord blood-derived exosomes that were loaded with miR-21-3p promoted fibroblasts function and angiogenesis for the improvement of wound healing [327]. In other study, miR-129 and -335 promoted wound healing in diabetic wounds by inhibiting the expression of Sp1-mediated MMP-9 expression [328]. Furthermore, miR-497 accelerated diabetic wound healing by decreasing the pro-inflammatory cytokines (IL-1β, IL-6, and TNF-α) and enhanced wound closure [329]. In another study, the inhibition of miR-133b resulted in accelerated wound healing in diabetes-impaired wound healing through the restoration of epidermal growth factor receptor (EGFR) expression and angiogenesis [330]. In a recent study, exosomal-cargoed miRs demonstrated a pivotal role in modulating angiogenesis in diabetic wound healing by regulating the expression of VEGF and downstream signaling transduction [303]. Although miR-based therapy has demonstrated promising results in in vivo studies for the improvement of chronic wound healing, it are still in its early phase for wound healing applications. The modulation of intricate and complex processes involving a network of interacting factors using single miR pose a considerable challenge. Nevertheless, a better understating and more exploration of miR functions and processes would be required to develop this approach as effective therapies for the treatment of different types of chronic wounds.

## 6. Challenges and Future Prospective

In recent decades, improvement in cutaneous wound healing remains a hot topic and has stimulated researchers to further explore the underlying mechanisms of wound healing and the development of newer therapeutic approaches for quality wound healing. Although the basic mechanisms of wound healing have been elucidated in several studies, certain specific mechanisms that are related to wound healing still remain unclear. In this context, several emerging and innovative strategies for improved and accelerated wound healing has been developed owing to tremendous advancements in science and technology. Currently, the nanotherapeutic-based approach has garnered much attention to promote wound healing and preventing scar formation. Nanotherapeutics employs a wide range of nanomaterials viz., nanoparticles, nano emulsions, nanofibers, nanogels, polymeric micelles, etc., either as a therapeutic device or drug delivery vehicle for offering stimulating effects during each phase of wound healing. Nevertheless, different nanomaterial-based strategies hold great potential for the treatment of different types of chronic wounds owing to their beneficial properties. However, there are still a few challenges that are associated with the utilization of nanotherapeutics for wound healing applications, which needs to be addressed beforehand. First and foremost, the challenge that is associated with nanotherapeutics includes biological safety of nanomaterial-based products as nanomaterials directly interact with the tissues that are present inside the wound. Therefore, transdermal toxicities risk is associated with nanomaterials that may cause allergies and skin irritation, suggesting the need for consideration of biological safety of nanomaterials before wound healing application [97]. The toxicities that are associated with the nanomaterials generally depends on the particle size, stability, shape, and its concentration [331]. In this context, the adjustment of physical and chemical properties of nanomaterials should be performed to reduce the associated skin toxicities while developing newer nanomaterials for wound healing. In addition, various stabilizers such as surfactants, metal shell, polymers, covalent adsorption, electrochemical synthesis strategy, and particle size control technology should be applied to reduce the skin irritation [332,333]. Another challenge includes the heterogenous nature of different chronic wounds which limits the regeneration potential and self-healing mechanisms of nanomaterials. In order to overcome this limitation, a better understanding of cellular cascades and the underlying mechanisms for the development of personalized nanomaterials for particular types of wounds would be necessary for wound healing. Additionally, newer strategies for addressing chronic wounds using nanotherapeutics employ emerging technologies without biologicals or drugs in order to reduce the fabrication cost and time to reach the market for clinical utility. Apart from these challenges, the cost of nanoformulations represents another limiting factor for nanomaterial utilization in clinical settings for wound healing. In order to overcome this limitation, there are various strategies being employed and explored such as a reduction in the dosage of nanomaterials, addition of low-cost adjuvants for the fabrication of composite nanomaterials [334], and the utilization of microneedles and layered self-assembly of nanomaterials for the controlled and sustained release of the loaded drugs [335,336]. Furthermore, optimization of the fabrication processes and nanoformulations would be an effective tool for the reduction of production costs. Another major challenge with nanotherapeutic-based approaches includes the differences in animals and human systems in terms of metabolic and functional properties. Most of the studies involving nanomaterials include animals such as mice, rats, and rabbits, which differ greatly in skin morphology and wound healing processes. Although large animals such as pigs and humans have much resemblance in terms of skin morphology and properties, they are still not widely utilized for wound healing applications. Nanomaterials are being utilized in different formulations using different drugs and methods in recent years, however, the mechanisms through which nanomaterials promote wound healing is superficially studied. The most common signaling pathways that have been studied for wound healing include the TGF-β1/SMAD signaling pathway (proliferation phase) and macrophage polarization (inflammatory phase) with respect to nanomaterials for the reconstruction of skin and improving wound healing [337]. Other related mechanisms using nanomaterials for wound healing have rarely been reported. As we are utilizing different types of nanomaterials with varying properties, there should be multifaceted mechanisms that are associated with the promotion of wound healing. Therefore, the exploration of innovative strategies for a deeper knowledge of mechanisms and improvement of treatment modalities is needed in future studies. In addition, standardized and controllable modeling methods are required to reflect the mechanisms of wound healing in different types of wounds. Despite the beneficial roles of nanomaterial-based therapy in all phases of wound healing, the same nanomaterial can’t be sufficient for all phases of wound healing as the requirements would vary greatly with passing phases of wound healing. In order to alleviate this issue, the requirement of real-time indicator is pertinent for the assessment of wound conditions. To attempt this, self-powered implantable electronic skins have been developed using ZnO nanowires that were modified by enzymes (urease and uricase) for the transcutaneous detection of human health by monitoring various parameters such as temperature, blood pressure, electrolyte metabolites, and humidity, etc. [338]. In addition, electronic skin would be able to monitor pH values, signaling pathway proteins, and inflammatory factors in the future with more standardization and optimization strategies. Taken together, the combination of nanotechnology and electronic skin have opened several new vistas for controlling wound healing treatment in real-time using appropriate nanomaterials.

Another promising strategy for improving wound healing includes the very promising and emerging technology, 3D bioprinting. 3D bioprinting techniques allows the precise deposition of skin structure and functional composition by a layer-by-layer approach directly on the wound itself through an in situ bioprinting technique without the requirement of any long-term incubation. In addition, a combination of 3D bioprinting, sensors, and imaging techniques would improve the wound healing outcomes and gradually lead towards precision medicine. For example, 3D bioprinted hydrogel-based dressing that is integrated with electronic components allows for the real-time monitoring of wound conditions [339]. Up till now, enormous success has been observed in the skin bioprinting filed, however, there still remains a few limitations. The major limitation that is related to skin bioprinting for wound healing is the time requirement to obtain autologous cells to fabricate skin constructs which has not been diminished sufficiently yet. Patients with extensive burn wounds require treatment in shorter time. Therefore, the current focus of skin bioprinting lies in the acceleration of wound recovery with a reduction of hypertrophic scar tissue.

Among cell therapies, stem cell-based therapies are the most common and effective for the promotion of wound healing using different strategies such as exploration of newer stem cells sources (induced pluripotent stem cells), utilization of stem cell-derived exosomes, genetic modifications of stem cells, etc. Despite the promising results of induced pluripotent stem cells (iPSCs) and iPSCs-derived exosomes for enhanced wound healing, there still remains a few challenges with the stem cell’s source, such as genetic instability, potential immunogenicity, infection risk, risk of carcinogenesis, and high processing costs [340]. In order to overcome immunogenicity and cancer risk, autologous iPSCs that are produced from non-viral vectors are being utilized. Taken together, extensive analysis for further understating the safety and reliability of iPSCs reprogramming technology is required for expand iPSC-based therapies for wound-healing. Furthermore, stem cell-derived exosomes with immunomodulatory and anti-inflammatory effects promote wound healing through the stimulation of skin cells (fibroblasts, keratinocytes, endothelial cells), rejuvenation of senescent cells, inhibition of apoptosis, reduction of scar formation, and enhancement of angiogenesis [32]. However, clinical applications of stem cell-derived exosomes for wound therapy are so far limited due to the inadequate availability of clinical grade exosomes for wound treatment. In this regard, advancements in GMP-compliant scaling-up technology in the future would enhance the clinical utility of stem cell-derived stem cells for wound healing. Other innovative strategies being employed for improving wound healing include ECM-based strategies, cold atmospheric plasma-based treatment, and platelet-rich-plasma therapy. Despite the promising effects of dECM-based strategies using different matrix formats, the clinical outcomes are variable that mainly relies on different factors viz., fabrication methods, efficacy of decellularization, and post-decellularization processing [243]. In addition, variation in the clinical outcomes may be attributed due to the cellular sources which vary greatly in their composition to recreate cellular niches. For example, stromal cell-derived ECMs contain large amounts of collagens, while prominently basement membrane structures (laminin) are present in endothelial cell-derived ECM [237]. Currently, the ECM-based regenerative approaches are mostly in preclinical trials and often combined with other skin therapies such as cell therapy or hydrogels-based drug delivery. Therefore, more understanding and knowledge regarding the different fabrication procedures, cell sources, and decellularization would be required to tap the potential of ECM-based strategies for chronic wounds healing and skin regeneration. Cold atmospheric plasma (CAP) therapy serves as a safe, rapid, non-invasive, and painless therapeutic approach for accelerating wound healing. The beneficial roles of CAP treatment for wound care include the generation of ROS and RNS for reducing bacterial burden, stimulation of faster migration of cells, and rapid wound healing with shortening of the inflammation phase in case of chronic wounds [341]. Cold plasma treatment holds great potential in the future for controlling and preventing infectious skin diseases in addition to the promotion of wound healing. As cold atmospheric plasma treatment is a relatively new approach in wound healing promotion, an in-depth study for the evaluation of each component of plasma, and development of improved plasma devices should be explored further in future studies to pave its way forward towards effective wound care. Furthermore, a PRP-based therapeutic approach for wound healing is mediated through the secretion of platelet-derived growth factors and cytokines by triggering cellular regenerative processes and modulating the immune system. The PRP-based therapeutic approach is a personalized and non-standardized medicine owing to differences in patient’s endogenous and exogenous factors. The quality of PRP varies with exogenous factors (cellular composition, anticoagulants) and endogenous factors (age, gender, nutrition, disease-associated systemic factors) [342]. Therefore, in order to understand the true impact of PRP-based treatment on wound healing, a more holistic vision considering the impacts of endogenous and exogenous factors would be required for future studies. This approach would allow to design more efficient and personalized therapy that is mediated by PRP for wound healing and skin regeneration.

## 7. Conclusions

Despite the extensive research and enhanced knowledge regarding wound treatment strategies for quality wound healing, the treatment of hard-to-heal chronic wounds and larger wounds still remains a major challenge in the skin regeneration and wound care domain. As multiple cellular and molecular mechanisms are associated with wound healing, no single treatment is sufficient for complete wound healing. To overcome these limitations and further advance the knowledge that is related to wound healing, various emerging and innovative treatment strategies have been explored in recent years such as nanotherapeutics, stem cell research, 3D bioprinting-based strategies, cold plasma therapy, and platelet-rich plasma therapy, etc. Herein, we have provided an overview of the different innovative strategies that are employed currently for improved wound healing along with their advantages and shortcomings. These approaches are either utilized alone or combined in order to provide faster wound healing. These emerging strategies and approaches for the acceleration of wound healing have opened several avenues and hopes for wound care in patients that are suffering from hard-to-heal complex wounds. Furthermore, challenges that are associated with these innovative approaches and future prospective has been discussed to augment the wound care and management. Taken together, advancements of various technologies and exploration of newer and innovative approaches holds great potential for quality wound healing.

## Figures and Tables

**Table 2:** A representative list of different stem cells-based therapies for accelerated wound healing.

Source of Stem Cells	Type of Wounds	Findings	Reference
Bone marrow-derived stem cells	Acute (full thickness wound)	Administration: intradermal and intravenous. Significant improvement in inflammation phase shortening, overexpression of proliferation markers (Ki67, CD71, and CD90), collagen deposition, and granulation tissue re-organization	[152]
Bone marrow-derived stem cells and their extracellular vesicles (EVs)	Acute (full thickness wound)	Administration: chitosan/collagen scaffold delivery system. Accelerated wound healing, enhanced collagen deposition	[153]
Bone marrow-derived stem cells	Chronic (diabetic wound)	Administration: subcutaneously. Improved collagen deposition and wound healing	[154]
Adipose-derived stem cells derived exosomes	Chronic (diabetic wound)	Upregulation and downregulation of specific micro RNAs (miRNAs), Inhibition of inflammation, modulation of PI3K/AKT signaling pathway	[155]
Adipose-derived stem cells	Chronic (full thickness burns wound)	Administration: 3D printed scaffold delivery system. Acceleration wound contraction, faster re-epithelialization and healing	[156]
Adipose-derived stem cells	Chronic (diabetic wound)	Administration: hydrogel delivery system. Enhanced neo-vascularization and accelerated wound closure	[157]
Hair follicles stem cells	Acute (full-thickness excisional wound)	Administration: intradermal injection. Shorter inflammation phase, function vascularization, enhanced re-epithelialization	[158]
Hair follicles stem cells	Chronic (venous leg ulcers)	Administration: direct application-hair skin graft. Significant reduction in ulcer area, improved healing	[159]
Hair follicles stem cells	Acute (full thickness skin wound)	Administration: direct application-hair skin graft. Overexpression of prostate cancer-upregulated long noncoding RNA 1 (PlncRNA-1), accelerated epidermal regeneration and wound healing	[160]
Induced pluripotent stem cells	Acute (full-thickness skin Wounds)	Administration: direct topical application. Expedited wound closure, enhanced collagen deposition	[161]
Induced pluripotent stem cell-derived exosomes	Chromic (diabetic ulcers)	Administration: direct. Enhanced migration and proliferation of fibroblasts, accelerated wound healing	[162]
Induced pluripotent stem cell-derived microvesicles	Chronic (burn wound)	Administration: Local transplantation. Accelerated wound closure, promotion of keratinocytes migration, increased re-epithelialization,	[163]

**Table 3:** A representative list of bio-inks and bioprinting methods for wound healing applications.

Biomaterial/Bioink/Cells	Bioprinting Method	Type of Wound	Findings	Reference
Fibrin and collagen hydrogel (Fibroblasts and keratinocytes)	In situ extrusion bioprinting	Acute (full thickness skin wound)	Rapid wound closure, reduced contraction, and accelerated re-epithelialization.	[215]
Fibrin hydrogel with gelatin, glycerol, and hyaluronic acid (Keratinocytes, melanocytes, fibroblasts, follicle dermal papilla cells, and microvascular endothelial cells, preadipocytes.)	Extrusion bioprinting	Acute (full thickness skin wound)	Accelerated wound closure, promotion of epidermal barrier formation, reduction in wounds contraction, remodeling of collagen	[223]
Gelatin/sodium alginate/gelatin methacrylate hydrogel (Dermal fibroblasts and epidermal keratinocytes)	Extrusion bioprinting	Acute (full thickness skin wound)	Reduced wound contraction and scarring, enhanced skin epithelialization, accelerated wound healing	[225]
Plasma-derived fibrinogen-containing factor XIII, fibronectin, thrombin, and macrophages (FPM bioink) (Primary fibroblasts human endothelial cells, and keratinocytes)	Extrusion bioprinting	Acute (full thickness skin wound)	Rapid wound closure and facilitation of re-epithelialization process	[226]
Fibrinogen/collagen hydrogel (Fibroblasts and keratinocytes)	In situ inkjet bioprinting	Acute (full thickness skin wound)	Improved wound closure and re-epithelialization process	[214]
Fibrin-collagen hydrogel (Amniotic fluid-derived stem (AFS) cells and bone marrow-derived mesenchymal stem cells (MSCs)	In situ extrusion bioprinting	Acute (full thickness skin wound)	Enhanced angiogenesis and wound closure rates	[227]
Skin-derived extracellular matrix (S-dECM) bio-ink (Fibroblasts, keratinocytes, endothelial progenitor cells and adipose-derived stem cells (ASCs)	Extrusion and inkjet bioprinting	Acute (full thickness skin wound)	Accelerated wound closure, enhanced re-epithelization, and neovascularization	[221]
Living photosynthetic microalgae scaffolds	In situ bioprinting	Chronic (diabetic wound)	Significantly reduced local hypoxia, accelerated chronic wound closure increased angiogenesis, and enhanced extracellular matrix (ECM) synthesis	[228]
Sodium alginate/gelatin/collagen hydrogel (Fibroblasts and keratinocytes)	Extrusion bioprinting	Acute (full thickness skin wound)	Enhanced re-epithelialization, reduced skin wound contraction, and accelerated wound healing	[229]
Strontium silicate (SS) microcylinders (Fibroblasts and keratinocytes)	Extrusion bioprinting	Acute and chronic wounds	Outstanding angiogenesis and wound healing	[230]

**Table 4:** Representative list of the cold atmospheric plasma treatment-based applications on chronic and acute wound healing.

Type of Wounds	Number of Patients	Type of Plasma Treatment/Device/Injected Gas/Exposure Time	Findings	Reference
Chronic	n = 36	MicroPlaSter cold plasma alpha device with argon (5 min daily treatment)	Significant bacterial load reduction (34%)	[295]
Chronic	n = 24	MicroPlaSter cold plasma alpha device, MicroPlaSter cold plasma beta device with argon (2 min daily treatment)	Significant reduction in bacterial load (40%)	[296]
Chronic	n = 70	MicroPlaSter cold plasma alpha device (3–7 min treatment)	Accelerated wound healing	[289]
Acute (wounds present at donor skin graft site)	n = 40	Cold atmospheric argon plasma, Plasma jet with argon (2 min every day for 1 week)	Improved re-epithelialization at donor sites	[297]
Acute (trauma)	n = 2	Plasma jet device with electrodes (20 min treatment)	Stopping of wound exudation, improved wound healing	[298]
Chronic (venous leg ulcers)	n = 14	PlasmaDerm^®^ VU-2010 device (45 s/cm^2^ for maximum 11 min thrice in a week for 8 weeks)	Strong antibacterial effects, significant reduction in chronic ulcer size	[299]
Chronic (venous leg ulcers)	n = 16	Antiseptic effects of cold atmospheric pressure plasma (APP) or octenidine (OCT) with argon	Significant microbial reduction (64%) without cytotoxicity	[300]
Chronic	n = 34	Tissue-tolerable plasma (TTP) and modern conventional liquid antiseptics	Provided most efficient strategy using antiseptic treatment, highest antimicrobial efficacy,	[301]
Chronic (pressure ulcers)	n = 50	Low-temperature atmospheric-pressure plasma (LTAPP) jet with argon (1 minute/cm^2^ once in a week for 8 weeks)	Reduction in bacterial load, significantly better PUSH (Pressure Ulcer Scale for Healing) score	[292]
Chronic (diabetic foot)	n = 65	Argon Plasma Jet (8 times treatment within 14 days)	Reduction in wound surface area, change in microbial load	[302]
Chronic (diabetic wounds)	n = 14	Cold atmospheric plasma	Promotion of vascularization, granulation tissue formation, and re-epithelialization	[286]

**Table 5:** A representative list of microRNAs (miRs) that are involved in different phases of wound healing.

Wound Healing Phase	microRNAs (miRs)	Function	Effect on Wound Healing	Reference
Inflammation	miR-142-3p/5p	Promotion of neutrophils migration	Promote wound healing	[311]
Inflammation	miR-203	Alleviation of skin inflammation	Promote wound healing	[312]
Inflammation	miR-23b	Escalation of of anti-inflammatory cytokines and reduction of pro-inflammatory cytokines	Promote wound healing	[313]
Inflammation	miR-27b	Reduce ROS production	Promote wound healing	[314]
Inflammation	miR-34	Enhancement of NF-κB signaling pathway activity	Impede wound healing	[315]
Angiogenesis	miR-615-5p	Inhibition of angiogenesis by targeting protein kinase B/endothelial nitric oxide synthase signaling pathway	Impede wound healing	[316]
Angiogenesis	miR-21	Suppression of angiogenesis by downregulating the expression of tensin homolog (PTEN) and SMAD7 genes	Impede wound healing	[317]
Angiogenesis	miR-126	Enhances migration and repair of endothelial cells	Promote wound healing	[318]
Angiogenesis	miR-221 and miR-222	The expression of endothelial NO	Impede wound healing	[319]
Re-epithelialization	miR-31	Enhances keratinocytes proliferation and migration	Promote wound healing	[320]
Re-epithelialization	miR-21	Promotes fibroblasts differentiation, collagen synthesis, and re-epithelialization	Promote wound healing	[321]
Granulation tissue formation	miR-29b	Inhibits expression of heat shock protein 47 (HSP47) and collagen synthesis	Impede wound healing	[322]
Granulation tissue formation	miR-185	Inhibits fibroblasts growth and function	Impede wound healing	[323]

## Data Availability

Not applicable.
